# Understanding the dynamic nature of plant lipid anabolic and catabolic metabolism is key to sustainable oilseed engineering

**DOI:** 10.1111/nph.70849

**Published:** 2025-12-24

**Authors:** Prasad Parchuri, Sean T. McGuire, Matthew G. Garneau, Niña Alyssa M. Barroga, Philip D. Bates

**Affiliations:** ^1^ Institute of Biological Chemistry Washington State University Pullman WA 99164 USA; ^2^ Division of Biology Kansas State University Manhattan KS 66506 USA

**Keywords:** acyltransferase specificity, fatty acid synthesis, lipases, lipid metabolic flux, lipid remodeling, oilseed engineering, triacylglycerol biosynthesis, unusual fatty acids

## Abstract

Plant‐derived oils are essential sources of reduced carbon and various fatty acid (FA) structures for food, biofuels, and the oleochemical industry. Despite extensive efforts, engineering mainstream oilseed crops to produce high levels of industrially valuable unusual FAs (UFAs) remains challenging. This review synthesizes recent advances in the understanding of lipid metabolic networks, emphasizing how species‐specific regulation of FA synthesis, activation, and delivery influences triacylglycerol (TAG) assembly to govern the efficiency of UFA accumulation. Key insights reveal that acyl flux through anabolic and catabolic branches of lipid metabolism is tightly controlled by enzyme substrate selectivities, diacylglycerol (DAG) pool compartmentalization, and metabolic context, including lipid remodeling and degradation pathways. Engineering success is often constrained by incompatibilities between UFA biosynthetic enzymes and endogenous host metabolism, leading to flux imbalances, futile cycles, and undesired phenotypes. We highlight emerging strategies to overcome these barriers, such as the use of UFA‐selective acyltransferases, coordinated manipulation of DAG source pools, suppression of competing endogenous enzymes, and exploitation of TAG remodeling mechanisms. This integrated synthesis provides a conceptual framework for logic‐based engineering of oilseeds with enhanced UFA content by offering new avenues for sustainable biomanufacturing of valuable lipids.

**Table:** 

	Contents	
	Summary	2196
I.	Introduction	2197
II.	Current engineering targets for controlling *de novo* FA biosynthesis for enhanced total oil accumulation and altered nascent FA composition	2199
III.	Activation and delivery of acyl groups for oil anabolism	2200
IV.	Oilseed TAG biosynthesis: a concert of anabolic and catabolic reactions	2201
V.	Modulation of lipid catabolism for enhanced oil bioengineering	2207
VI.	Conclusion and future outlook	2209
	Acknowledgements	2209
	References	2210

## I. Introduction

Plant seed oils primarily composed of triacylglycerols (TAGs) are a renewable source of reduced carbon and stored energy. Fatty acids (FAs) within plant TAGs are vital for human nutrition, providing 25% of caloric intake and essential polyunsaturated FAs (PUFAs) required in the diet (Zhou *et al*., 2023). Seed oils are also widely used as feedstocks in the oleochemical industry for biofuels (biodiesel, renewable diesel, and sustainable aviation fuels), polymers, lubricants, paints, cosmetics, soaps, and pharmaceuticals (Dyer *et al*., 2008; Ohlrogge *et al*., 2018). Global vegetable oil production reached a record 218 million metric tons in 2023 and is projected to continue to rise (USDA, 2025). However, an increasing share (28% of global production) of vegetable oil production is being diverted to biofuels and renewable diesel, raising concerns about food security and land use (Meijaard *et al*., 2020), thus necessitating further research to both increase oil yields and produce optimized FA compositions for various end uses.

Oils from the major plant oil crops (Table 1) contain predominantly five FAs in varying proportions: saturated FAs, palmitic acid (16:0) and stearic acid (18:0); the monounsaturated FA, oleic acid (18:1); and the PUFAs, linoleic acid (18:2 n‐6) and alpha‐linolenic acid (18:3 n‐3). Beyond these common FAs, *c*. 450 unusual FAs (UFAs) have been identified across various plant species (Table 1). UFAs differ from conventional FAs in their chain length (< 16 or > 18 carbon atoms); methyl branching; number, position, and unsaturation configuration (e.g. double, triple, or conjugated double bonds); or the presence of additional functional groups, such as hydroxyl, keto, epoxy, or cyclic structures (e.g. cyclopropane, cyclopentene, and phenyl; Ohlrogge *et al*., 2018). The end use of each vegetable oil for food or industrial purposes depends on the FA composition. Oils rich in oleic acid are more oxidatively stable at high temperatures, making them ideal for cooking, whereas oils high in saturated FAs are better for baking and PUFAs are preferred for margarine, spreads, and salad dressings (Dyer *et al*., 2008). Furthermore, an omega‐6/omega‐3 (n‐6/n‐3) FA ratio of 1 : 1 to 4 : 1 is ideal in human diets to prevent chronic diseases and reduce the production of pro‐inflammatory eicosanoids (Simopoulos, 2003; Prasad *et al*., 2021). Structural variations of UFAs in seed oils confer unique chemical properties, making them valuable feedstocks for biofuel and oleochemical industries. For instance, medium‐chain FAs (MCFAs), such as lauric acid (12:0), from coconut and palm oil are key ingredients in soaps and detergents. MCFAs and palmitoleic acid (16:1) are also used in aviation biofuel production due to their chemical similarity to petroleum‐derived alkanes of equivalent carbon chain lengths (Kallio *et al*., 2014; Esfahanian *et al*., 2021). Very‐long‐chain monounsaturated FAs (VLCFAs), including eicosenoic acid (20:1) and erucic acid (22:1) from meadowfoam (*Limnanthes alba*), crambe (*Crambe abyssinica*), *Brassica napus*, and *Brassica carinata* are widely used in cosmetics, lubricants, and plastic films (McKeon *et al*., 2016). Conjugated FAs, such as those found in tung oil, are used in paints and coatings, while hydroxy FAs (HFAs) like ricinoleic acid from castor (*Ricinus communis*) and lesquerolic acid from *Physaria* are valuable for producing lubricants, cosmetics, and polymers (Ohlrogge *et al*., 2018). However, most UFA‐producing species possess poor agronomic traits, such as geographical and climatic limitations, small seed size, low yield, seed shattering, tree/shrub growth habits, and heterogeneous populations, thus making large‐scale cultivation challenging or requiring deforestation to cultivate oilseed tree species, such as palm (Voelker & Kinney, 2001; Dyer *et al*., 2008; Meijaard *et al*., 2020). Additionally, some UFA‐producing crops may produce toxic compounds that are undesirable by‐products, such as the ricin in castor seeds (Severino *et al*., 2012). Therefore, to meet the growing demand for plant oils containing select FAs, we need to identify the differential enzymatic mechanisms that control FA composition and oil accumulation from species producing FAs of interest and in the crops that will be platforms for future bioengineering. Understanding how plants produce unique plant oils will enable rational design of oilseed crops to meet the rising and diverse demands for their societal use. This review focuses on understanding lipid metabolism relevant to bioengineering UFAs into seed oils as feedstocks for the chemical and biofuel industries. Successes in bioengineering healthy omega‐3 FA from algae into seed oils or production of wax esters have been reviewed elsewhere (Venegas‐Calerón *et al*., 2010; Domergue & Miklaszewska, 2022; Napier & Betancor, 2023).

**Table 1:** Fatty acid profile of common oilseed crops and other plants with unusual fatty acids (UFAs).

Plant species	8:0	10:0	12:0	14:0	16:0	18:0	18:1	18:2	18:3	20:0	20:1	18:1‐epoxy	18:1‐OH	18:2‐OH	20:1‐OH	20:2‐OH	18:3‐conj	17:0‐CPA	19:0‐CPA	22:1	22:2
**Fatty acid profile (%) of common oilseeds**																					
Avocado (*Persea gratissimima*)	–	–	–	–	18.0	0.4	62.1	11.0	0.5	–	–	–	–	–	–	–	–	–	–	–	–
High oleate Canola (*Brassica napus*)	–	–	–	–	3.9	1.6	60	22	10.1	–	–	–	–	–	–	–	–	–	–	–	–
Cocoa (*Theobroma cacao*)	–	–	–	–	25.1	36.4	34.1	2.8	0.2	–	–	–	–	–	–	–	–	–	–	–	–
Coconut (*Cocos nucifera*)	–	7.8	50.5	18.5	6.8	2.3	5.6	1.8	0.1	–	–	–	–	–	–	–	–	–	–	–	–
Cottonseed (*Gossypium hirsutum*)	–	–	–	–	27.1	2.33	17.8	43.1	0.1	–	–	–	–	–	–	–	–	–	0.32	–	–
Olive (*Olea europaea*)	–	–	–	–	11.5	2.5	75.5	7.5	1	–	–	–	–	–	–	–	–	–	–	–	–
Palm kernal (*Elaeis guineensis*)	6.9	7.2	42.7	15.9	9.0	1.9	13.0	3.4	–	–	–	–	–	–	–	–	–	–	–	–	–
Peanut (*Arachis hypogea*)	–	–	–	–	13.7	3.2	41.1	37.6	–	1	0.8	–	–	–	–	–	–	–	–	–	–
Soybean (*Glycine max*)	–	–	–	–	7	5.5	26.1	54.7	5.8	–	–	–	–	–	–	–	–	–	–	–	–
**Fatty acid profile (%) of plants with unusual fatty acids (UFA)**																					
Bitter gourd (*Momordica charantia*)	–	–	–	–	1.5	17.4	14.6	8.6	–	–	–	–	–	–	–	–	56.2	–	–	–	–
Castor (*Ricinus communis*)	–	–	–	–	7.77	2.87	4	4.8	0.8	–	–	–	88.8	–	–	–	–	–	–	–	–
Crambe (*Crambe abyssinica*)	–	–	–	–	2.12	0.88	19.4	8.93	5.8	1.63	3.87	–	–	–	–	–	–	–	–	57.1	–
Cuphea (*Cuphea viscosissima*)	–	75.5	3	1.3	3.1	0.3	1.9	4.7	0.5	0.3	0.4	–	–	–	–	–	–	–	–	–	–
Cuphea (*Cuphea hookeriana)*	50.2	25.2	3.6	1	7.1	0.7	4.3	4.6	0.4	–	–	–	–	–	–	–	–	–	–	–	–
Cuphea (*Cuphea koehneana*)	–	91.6	1.5	0.6	1.3	0.3	1.1	3.1	0.2	0.1	0.1	–	–	–	–	–	–	–	–	–	–
Cuphea (*Cuphea pulcherrima*)	94.4	3.3	–	–	0.6	–	0.7	1	–	–	–	–	–	–	–	–	–	–	–	–	–
Ironweed (*Vernonia galamensis*)	–	–	–	–	2.7	3.2	6.3	14.5	–	–	–	72.8	–	–	–	–	–	–	–	–	–
Lychee (*Litchi chinensis*)	–	–	–	0.9	12.1	5.1	27.7	1.2	4.5	0.6	–	–	–	–	–	–	–	4	40.9	–	–
Meadowfoam (*Limanthes alba*)	–	–	–	–	0.6	–	1.8	2.5	–	0.7	62.8	–	–	–	–	–	–	–	–	12.3	15.7
*Physaria (Physaria fendleri)*	–	–	–	–	1.5	2.2	15.5	9.9	11.9	–	1.1	–	–	–	53.5	3.1	–	–	–	–	–
Pennycress (*Thlaspi arvense*)	–	–	–	–	3.9	3.7	14.5	26.3	12.2	–	11	–	–	–	–	–	–	–	–	31	–
Tung tree (*Vernicia fordii*)	–	–	–	–	2.68	2.42	6.35	8.19	0.93	0.19	–	–	–	–	–	–	79.1	–	–	–	–

Dash (−) indicates no fatty acid data available. All information is obtained from PlantFAdb (Ohlrogge *et al*., 2018).

Recently, new oilseed crops like *Buglossoides arvensis*, *Thlaspi arvense* (pennycress), and *Camelina sativa* (*Camelina*) have been developed for food and industrial use by classical and molecular breeding approaches due to their unique FA profiles, short life cycle, and potential as winter cover crops (Zanetti *et al*., 2013; Cumberford & Hebard, 2015; Prasad *et al*., 2019; Chopra *et al*., 2020). Additionally, various bioengineering strategies have been explored over the past two decades to overcome agronomic challenges and enhance seed oil content or modify FA composition in engineered oily plants. However, successful production of very high levels of FA of interest has been limited (Yu *et al*., 2019; Lunn *et al*., 2021; Sun *et al*., 2022), with only a few exceptions (Nguyen *et al*., 2010; Han *et al*., 2020; Alkotami *et al*., 2024). For instance, genetic engineering techniques, such as CRISPR‐Cas9 and RNA interference (RNAi), have effectively optimized levels of common endogenous FAs (mostly 18:1) in crops like soybean, *Camelina*, canola, peanut, pennycress, safflower, and cotton (Zhou *et al*., 2023). In contrast, bioengineering efforts to produce UFAs, such as MCFAs, HFAs, epoxy, and cyclopropane FAs (CPFAs) in *Arabidopsis*, *Camelina*, soybean, and pennycress have faced challenges. These attempts often incur seed oil penalties, including low yields of target FAs, impaired seed germination, and reduced plant growth and yield (Smith *et al*., 2003; Lu *et al*., 2006; Li *et al*., 2010; Bates *et al*., 2014; Yu *et al*., 2018; Esfahanian *et al*., 2021; Lunn *et al*., 2021). Although most major enzymes involved in UFA synthesis and glycerolipid assembly have been identified, the limited success in achieving high levels of UFAs highlights that critical knowledge gaps remain. Specifically, it is unclear how to engineer crops to boost total oil, accumulate UFAs in seed TAGs without affecting membrane function, or prevent host species from degrading the produced UFA. Addressing these gaps is essential for advancing the development of engineered oily plants with enhanced agronomic and industrial traits.

## II. Current engineering targets for controlling *de novo* FA biosynthesis for enhanced total oil accumulation and altered nascent FA composition

Plant FA synthesis (FAS) has been engineered at multiple levels to modify oil yield and modify FA composition. Approaches have included redirecting carbon flux toward oil accumulation (e.g. overexpression of *Wrinkled1* transcription factor) or channeling FA precursors into plastids with varying degrees of success (Lee *et al*., 2011; Tang *et al*., 2022a,b; Sagun *et al*., 2023; Mukherjee *et al*., 2025). However, direct regulation of the FAS rate and the specific FAs produced in plastids remains key targets for engineering seed oil accumulation and tailoring the oil FA composition.

### 1. Optimizing heteromeric ACCase to increase FA synthesis

**Fig. 1:**
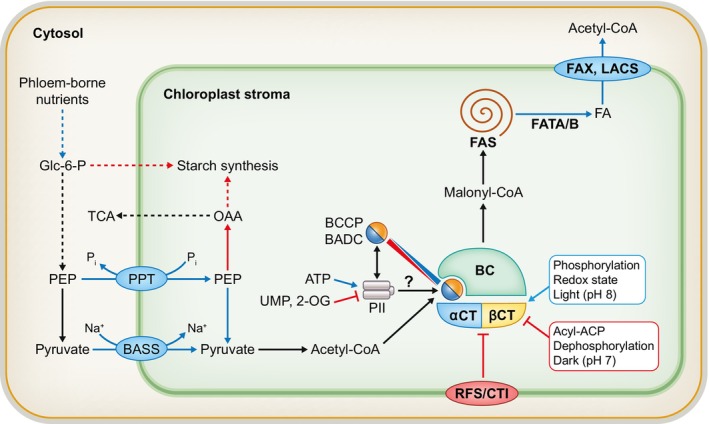
Regulatory factors affecting seed oil accumulation and heteromeric acetyl‐CoA carboxylase (ACCase) activity. The diagram shows characterized factors that affect fatty acid synthesis including substrate production, heteromeric ACCase (htACCase) activity, and FAS. Enzymes and pathways that positively affect FAS when overexpressed or upregulated are denoted in blue, while pathways with negative effects are labeled in pink (with blunt arrows), dashed lines indicate multiple enzymatic steps. Modifications to nutrient delivery as well as phosphoenolpyruvate and pyruvate delivery transport into plastids via phosphoenolpyruvate/phosphate translocator (PPT1) and bile acid: sodium symporter family protein 2 (BASS2) had positive effects on seed oil accumulation. For model purposes, the BC : BCCP : BADC ratio was assumed to be 1 : 1 : 1. The association between BCCP and BADC proteins is shown to have a rheostat‐like effect on ACCase activity regulated in part through diurnal changes in the ionic milieu as well as interaction with the cellular energy and nitrogen sensor PII. Regulation of the carboxyltransferase complex (α‐CT/β‐CT), half of the heteromeric ACCase, is summarized to the right including phosphorylation, reduction in sulfide bonds, feedback inhibition via acyl‐ACP, and diurnal responses to light via pH. The CT complex also associates with the inner envelope RFS and CTI proteins to inhibit ACCase activity. Following malonyl‐CoA production and FA synthesis via FAS, acyl‐ACPs are cleaved to free fatty acids via the FATA/B thioesterases, which are also targets of oil/FA engineering. Final export of free fatty acids and catalysis to acyl‐CoA substrates via the FAX and LACS proteins are also targets of increased oil engineering. 2‐OG, 2‐oxoglutarate; BADC, biotin/lipoyl attachment domain‐containing protein; BASS, bile acid sodium symporter; BC, biotin carboxylase; BC, biotin carboxylase; BCCP, biotin carboxyl carrier protein; BCCP, biotin carboxyl carrier protein; CT, carboxyltransferase; CTI, carboxyltransferase interactor; FAS, fatty acid synthase complex; FAT, fatty acyl‐ACP thioesterase; FAX, fatty acid export; glc‐6‐P, glucose‐6‐phosphate; LACS, long‐chain acyl‐CoA synthetase; OAA, oxaloacetate; PEP, phosphoenolpyruvate; PPT, PEP/Pi Transporter; RFS, regulator of fatty acid synthesis; α/β‐CT, carboxyltransferase.

The first committed step of FAS is the carboxylation of acetyl‐CoA to malonyl‐CoA by acetyl‐CoA carboxylase (ACCase) in the plastid. In monocots, ACCase is a single protein containing multiple functional domains similar to animals (Konishi *et al*., 1996; Niazi & Moorhead, 2025). While dicots predominantly utilize a heteromeric ACCase (htACCase) complex similar to bacteria that includes catalytic, structural, and regulatory subunits that affect complex assembly, stability, localization, and activity (Salie & Thelen, 2016; Ye *et al*., 2020a,b; Zhou *et al*., 2024; Niazi & Moorhead, 2025). The core htACCase complex includes the subunits biotin carboxylase (BC), biotin carboxyl carrier protein (BCCP), and α‐ and β‐carboxyltransferase (CT). ACCase enzymes (homomeric and heteromeric) are regulated by a variety of factors including pH, redox status, feedback inhibition, and phosphorylation (Fig. 1; Huerlimann & Heimann, 2013; Salie & Thelen, 2016; Ye *et al*., 2020a; Zhou *et al*., 2024). However, htACCase is also controlled through holoenzyme assembly where α‐CT and biotinylated BCCP can be limiting (Reverdatto *et al*., 1999; Ke *et al*., 2000; Thelen *et al*., 2001; Thelen & Ohlrogge, 2002b; Gu *et al*., 2011; Wilson & Thelen, 2018; Wang *et al*., 2022). Additionally, htACCase holoenzyme assembly and activity are influenced via multiple regulatory proteins (Keereetaweep *et al*., 2018; Wilson & Thelen, 2018; Ye *et al*., 2020a; Zhou *et al*., 2024). The α‐CT subunit can be sequestered to the inner chloroplast envelope by carboxyltransferase interactor (CTI) and regulation of FA synthesis (RFS) proteins which reduces ACCase activity and FAS (Thelen & Ohlrogge, 2002a; Ye *et al*., 2020a; Zhou *et al*., 2024). Biotin/lipoyl attachment domain‐containing proteins (BADC) are similar to BCCP's but lack the biotin attachment domain (Keereetaweep *et al*., 2018). While these proteins were initially described as negative regulators (Keereetaweep *et al*., 2018; Liu *et al*., 2019), recent evidence suggests that they may participate in diurnal regulation (BADC1 and 2; Ye *et al*., 2020b; Kim *et al*., 2023) and promote htACCase complex assembly by mediating interactions between BC and BCCP (BADC2 and BADC3; Shivaiah *et al*., 2020, 2025), suggesting a more nuanced and conditional role of BADC proteins. BADC and BCCP proteins are also co‐regulated by PII which likely modulates htACCase activity in response to N metabolism for the co‐regulation of seed oil and protein accumulation (Fig. 1; Feria Bourrellier *et al*., 2010; Garneau *et al*., 2025).

Ongoing research seeks to characterize the role of nonenzymatic regulatory proteins (BADC, PII, CTI, and RFS), their biological significance, and their potential to improve oil production and modify FA composition. For instance, the leaves of *cti1*, *2*, *3* and *rfs1* knockout lines increase FAS, lipid accumulation, and decrease FA desaturation. However, the seed oil phenotype was limited to reduced desaturation in only the *cti1* and *rfs1* lines or stacked *cti/rfs* knockouts (Ye *et al*., 2020b; Zhou *et al*., 2024). Conversely, *badc* knockout plants have been associated with modest effects on leaf metabolism but can have significant effects on seed FA composition and oil accumulation dependent on growth conditions, such as light and N input (Keereetaweep *et al*., 2018; Liu *et al*., 2019; Shivaiah *et al*., 2020; Ye *et al*., 2020a; Garneau *et al*., 2025). However, the *badc2/3* mutant is embryo lethal (Keereetaweep *et al*., 2018). Together, these results suggest that modulation of ACCase may be one approach to control FAS and thus total seed or leaf lipid content. To further optimize ACCase catalytic activity, future engineering strategies may benefit from an enhanced understanding of the exact function of each ACCase regulatory subunit under different cellular states and/or growth conditions. This is especially important when targeting leaf vs seed oil accumulation, as different regulatory mechanisms may dominate in each tissue type. Tissue‐specific gene expression modifications may be required to enhance ACCase activity while minimizing negative effects on plant development.

### 2. Modulating *de novo* FAS output to produce various UFA

FAs are synthesized 2‐carbons at a time (generally up to 16‐ or 18‐carbons) as acyl‐acyl carrier protein (ACP) esters (Zhang *et al*., 2024). The saturated 16:0‐ACP and 18:0‐ACP or monounsaturated 18:1Δ^9^‐ACP (produced by stearoyl‐ACP desaturase, SAD) are the major products of FAS in most plant cells. SAD proteins are also targets for the engineering of UFAs with alternative double bond locations (Nguyen *et al*., 2010; Dvorianinova *et al*., 2023; Wang *et al*., 2024). For extra‐plastidial membrane and TAG production, free FAs are cleaved from ACP by FA thioesterases (FAT) and exported from the plastid (Fig. 1; Koo *et al*., 2004; Kalinger & Rowland, 2023). FAT activity ultimately determines the FA length and which FAs are exported to the cytosol/endoplasmic reticulum (ER), and in some species, are key to the accumulation of MCFAs in seed oils.

A diverse range of thioesterases can be found in nature that produce FAs with chain lengths less than 16 carbons. FatB‐type thioesterases from *Umbellularia californica* (California bay laurel) and *Cuphea* spp. preferentially produce MCFAs 6–14 carbons in length (Filichkin *et al*., 2006; Tjellström *et al*., 2013; Kim *et al*., 2015b; Kalinger & Rowland, 2023). However, engineering plant‐based systems for increased MCFA production often presents challenges. MCFAs, such as octanoic acid (8:0) and decanoic acid (10:0), can disrupt membrane integrity and fluidity, leading to reduced cell viability and metabolic stress. To counteract this, strategies such as co‐expression of MCFA‐specific lipid metabolic enzymes (discussed in detail below, see Section IV) to rapidly convert free FAs into storage lipids have been demonstrated in *Arabidopsis*, *Camelina*, pennycress, and *Nicotiana benthamiana* (Reynolds *et al*., 2015; Kim *et al*., 2015a; Iskandarov *et al*., 2017; Bansal *et al*., 2018; Esfahanian *et al*., 2021). Despite this, engineering efforts produced MCFA only as a minor portion of total FAs with concurrent reductions to oil yield (Tjellström *et al*., 2013; Kim *et al*., 2015a; Iskandarov *et al*., 2017; Bansal *et al*., 2018; Esfahanian *et al*., 2021). Overall, the use of thioesterases in biofuel and bioplastic engineering has shown promising results, but further refinements in metabolic engineering approaches, including dynamic regulatory systems and compartmentalized FA processing, are necessary to optimize production while minimizing negative impacts on cellular health. Results of engineering different unusual FA compositions into seed oils (including MCFAs) are summarized in Supporting Information Table S1.

## III. Activation and delivery of acyl groups for oil anabolism

Free FAs exported from the plastid are activated for anabolism by esterification to CoA by long‐chain acyl‐CoA synthetases (LACS; Fig. 1; Schnurr *et al*., 2004). The LACS genes in 122 plant species are split into six major clades that have differential expression patterns (Ayaz *et al*., 2021). Of the nine LACS isoforms in *Arabidopsis*, distinct sub‐cellular localizations may contribute to differential roles in lipid metabolism (Shockey *et al*., 2002; Shockey & Browse, 2011). For example, LACS9 is located on the outer chloroplast envelope and LACS4 is ER‐localized (Jessen *et al*., 2014). Both *in vitro* and *in vivo* analyses indicate that LACS have various chain length specificities in both plants and microalgae (Jessen *et al*., 2014; Xu *et al*., 2018; Zhao *et al*., 2019; Bai *et al*., 2022), suggesting that LACS can contribute to controlling the FA composition of lipids. During seed development, LACS function redundantly for oil biosynthesis. Notably, the *lacs1/lacs9*, *lacs4/lacs9*, and *lacs4/lacs8* double mutant seed contains less oil than wild‐type (Zhao *et al*., 2010, 2019). Recently, *Physaria fendleri LACS4* and *LACS8* were introduced into *Arabidopsis* pre‐engineered to produce the HFA, ricinoleic acid (18:1‐OH; Bengtsson *et al*., 2022). Genes from *P. fendleri* were chosen due to the plant's ability to accumulate > 60% hydroxylated oils (Bhandari & Bates, 2021). Surprisingly, the *P. fendleri* LACS did not enhance HFA accumulation (Bengtsson *et al*., 2022). This result emphasizes that knowledge gaps remain in: identifying which endogenous enzymes are bottlenecks to accumulation of target FAs; predicting the FA selectivity of LACS (or other enzymes) from conservation of sequence identity alone, particularly from those species that accumulate UFAs; and understanding how exogenously expressed genes may or may not integrate into host plant metabolism to enhance target product accumulation.

Coincident with LACS activity is the binding of acyl‐CoAs to acyl‐CoA‐binding proteins (ACBPs). To date, the knowledge about ACBPs is limited to plants that accumulate common FA, namely, *Arabidopsis*, rice, and other Brassicas (Yurchenko *et al*., 2009; Du *et al*., 2016). ACBPs participate in anabolic reactions by delivering acyl‐CoAs to the site of biochemical synthesis (Li & Chye, 2003; Chen *et al*., 2008, 2010; Xiao *et al*., 2008) and have differential binding affinities for various acyl‐CoA esters (Hsiao *et al*., 2014). Of note, ACBP1/2 are localized to the ER and are required for seed development as the double mutant *acbp1/2* is embryonically lethal (Chen *et al*., 2010). A triple knockout of the cytosolic *ACBP4/5/6* causes pollen abortion and reduced seed set (Hsiao *et al*., 2014), while *acbp6‐1* mutant plants have greater 18:2 content at the expense of 20:1 in their seed oil (Guo *et al*., 2019). As most of the acyltransferases involved in both *de novo* diacylglycerol (DAG) and TAG synthesis are acyl‐CoA dependent, they rely on efficient delivery of acyl‐CoAs for catalysis. In the green alga, *Chromochloris zofingiensis*, fusion of *Arabidopsis* ACBP6 with DGAT1 enhances both the kinetic parameters of acyl transfer and total protein abundance (Xu *et al*., 2020). Although most ACBP amino acid sequences are evolutionary conserved (Færgeman *et al*., 2007), the array of natural FA variation has likely caused specialized lineages to adapt these proteins to effectively bind UFAs. Future research should address whether ACBP acyl‐CoA binding specificity and select protein–protein interactions with lipid biosynthetic acyltransferases are key for controlling seed oil compositions.

## IV. Oilseed TAG biosynthesis: a concert of anabolic and catabolic reactions

**Fig. 2:**
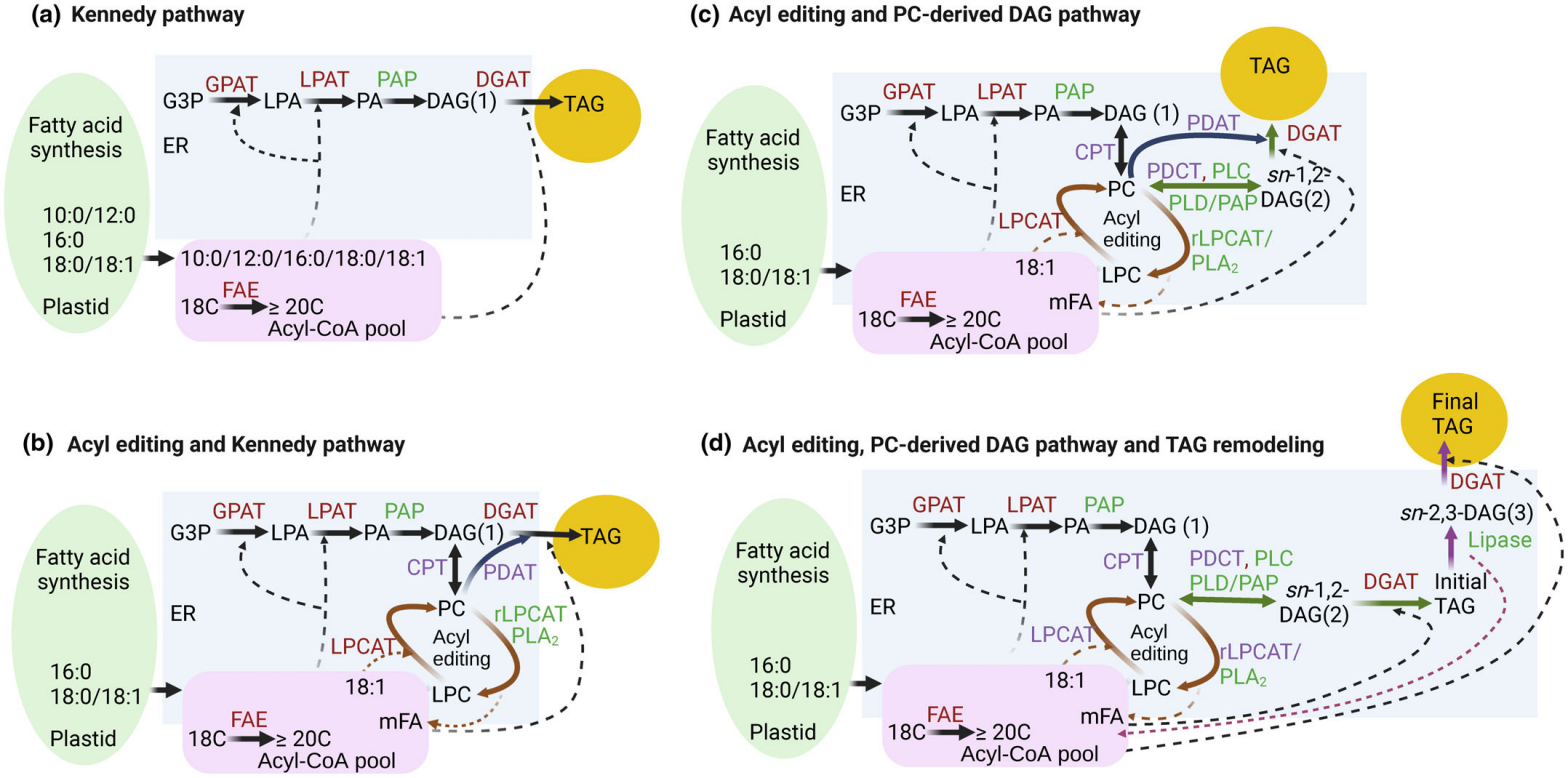
Diverse pathways of TAG biosynthesis in plants. (a) Linear Kennedy pathway; (b) acyl editing and Kennedy pathway; (c) acyl editing and PC‐derived DAG pathway of TAG synthesis; (d) acyl editing and PC‐derived DAG pathway of TAG synthesis and TAG Remodeling. Black arrows indicate the reaction of *de novo* DAG and TAG synthesis by the linear Kennedy pathway, brown, green, and purple arrows indicate reactions of acyl editing, PC‐derived DAG synthesis, and TAG remodeling, respectively. The blue arrow in (b) and (c) represents Acyl‐CoA independent TAG synthesis. Anabolic, catabolic, and amphibolic reaction catalyzing enzymes are represented in dark red color, green color, and purple color, respectively. Substrates are represented in black color. Fatty acids are represented as the number of carbon:double bonds (10:0/12:0 etc.). Dashed lines indicate acyl transfer or hydrolysis reactions. Substrate abbreviation: DAG, diacylglycerol; G3P, glycerol‐3‐phosphate; LPA, lysophosphatidic acid; LPC, lysophosphatidylcholine; mFA, PC‐modified FA; PA, phosphatidic acid; PC, phosphatidylcholine; TAG, triacylglycerol. Enzyme abbreviations: DGAT, acyl‐CoA:DAG acyltransferase; CPT, CDP‐choline:DAG cholinephosphotransferase; GPAT, acyl‐CoA:G3P acyltransferase; LPAT, acyl‐CoA:LPA acyltransferase; LPCAT, acyl‐CoA:LPC acyltransferase; PAP, PA phosphatase; PDAT, phospholipid:DAG acyltransferase; PDCT, PC:DAG cholinephosphotransferase; PLA_2_, phospholipase A2; PLC, phospholipase C; PLD/PAP, phospholipase D/PAP. DAG1, DAG2, and DGA3 represent different pools of DAG. *sn*‐1,2 and *sn*‐2,3‐DAG represent enantiomers of DAG. Created in BioRender. Bates, P. (2025) https://BioRender.com/b9qk6lt.

TAG biosynthesis in seeds is a complex process that overlaps with membrane lipid metabolism and involves a network of interconnected anabolic and catabolic reactions (Bates & Shockey, 2025; Fig. 2). *In vivo* isotopic labeling studies have revealed significant interspecies variation in acyl fluxes through the lipid metabolic network, contributing to the diversity of seed TAG FA compositions (Table 2). Plants accumulating predominantly *de novo* synthesized FAs (e.g. 8:0 to 18:1), such as *Cuphea*, avocado, cocoa, oil palm, and olive primarily, use the Kennedy pathway to assemble TAG in the ER (Fig. 2a; Table 2; Weiss *et al.*, 1960; Griffiths *et al*., 1988; Bafor *et al*., 1990; Bafor & Stymne, 1992; Ramli *et al*., 2002). The Kennedy pathway involves sequential acylation of glycerol‐3‐phosphate by glycerol‐3‐phosphate acyltransferase (GPAT) and lysophosphatidic acid acyltransferase (LPAT) to produce phosphatidic acid (PA) followed by dephosphorylation by phosphatidate phosphatase (PAP) to form *de novo* diacylglycerol (DAG 1), and final acylation by diacylglycerol acyltransferase (DGAT) to produce TAG (Fig. 2a; Bates & Shockey, 2025). However, many oilseeds such as *Arabidopsis*, rapeseed, *Camelina*, castor, *Physaria*, and soybean accumulate TAGs enriched in FAs modified by either elongation in the acyl‐CoA pool or by membrane lipid‐based addition of functional groups predominantly while esterified to ER‐localized phosphatidylcholine (PC). PC‐modified FAs that accumulate in seed TAG include membrane‐compatible PUFAs and membrane‐incompatible UFAs, such as hydroxylated, epoxidized, or conjugated FAs (Table 1). Their incorporation into TAG requires a complex interplay of lipid anabolic and catabolic reactions and FA flux through different species‐specific branches of the lipid metabolic network (Fig. 2b–d; Bates *et al*., 2009; Bates & Browse, 2011; Guan *et al*., 2014; Haslam *et al*., 2016; Bhandari & Bates, 2021; Pollard & Shachar‐Hill, 2022).

**Table 2:** Triacylglycerol synthesis from *de novo* diacylglycerol (DAG) or phosphatidylcholine (PC)‐derived DAG in seed tissue of various plants.^1^

Plant	WT FA composition *De novo* FA, 18:2 + 18:3, VLCFA	Initial Glycerol labeling PC : TAG ratio	Mutant/RNAi enzyme (% Δ PUFA, % Δ oil content)
***De novo* DAG‐based TAG synthesis via Kennedy pathway**			
Coriander (*Coriandrum sativum*)	86, 13, 0 (Cahoon & Ohlrogge, 1994)	VL: 1 (Cahoon & Ohlrogge, 1994)	
Cigar flower (*Cuphea lanceolata*)	89, 5, 0 (Dyer *et al*., 2008)	1 : 32 (Bafor *et al*., 1990)	
Oil palm (*Elaeis guineensis*)	92, 8, 0 (Dyer *et al*., 2008)	0 : 1 (Ramli *et al*., 2002)	
Olive (*Olea europaea*)	78–96, 4–21, 0–1 (Gunstone *et al*., 2007)	0 : 1 (Ramli *et al*., 2002)	
Avocado (*Persea americana*)	84, 16, 0 (Gaydou *et al*., 1987)	1 : 23 (Griffiths *et al*., 1988)	
Cocoa (*Theobroma cacao*)	98, 2, 0 (Griffiths & Harwood, 1991)	VL: 1 (Griffiths & Harwood, 1991)	
**PC‐derived DAG‐based TAG synthesis**			
Thale cress (*Arabidopsis thaliana*)	27, 48, 22 (Li‐Beisson *et al*., 2013)	14 : 1 (Bates & Browse, 2011)	PDCT (−40%, 0) (Lu *et al*., 2009) NPC6 (−4–8%, −5–7%) (Cai *et al*., 2020)
High oleate Canola (*Brassica napus*)	66, 31, 0 (Dyer *et al*., 2008)		PDCT (−18%, 0) (Bai *et al*., 2022)
High erucic Rapeseed (*Brassica napus*)	21, 23, 46 (Dyer *et al*., 2008)	3 : 1 (Guan *et al*., 2014)	NPC6 (genomic association to oil content) (Cai *et al*., 2020)
Gold of pleasure (*Camelina sativa*)	23, 52, 19 (Zubr & Matthäus, 2002)	1 : 0 (Pollard & Shachar‐Hill, 2022) 4 : 1 (Yang *et al*., 2017)	
Safflower (*Carthamus tinctorius*)	13, 87, 0 (Ichihara & Noda, 1980)	3 : 1 (Griffiths *et al*., 1988)	
Abyssinia (*Crambe abyssinica*)	15, 14, 66 (Li *et al*., 2012)	1 : 2 (Guan *et al*., 2014)	PDCT (−23%, n.d.) (Guan *et al*., 2015)
Soybean (*Glycine max*)	38, 62, 0 (Dyer *et al*., 2008)	49 : 1 (Bates *et al*., 2009)	PDCT (−50%, 0) (Lee *et al*., 2011) PLD (−20%, 0) (Lee *et al*., 2011)
Flax seed (*Linum usitatissimum*)	27, 71, 0 (Dyer *et al*., 2008)	5 : 1 (Slack *et al*., 1978)	
Fendler's bladderpod (*Physaria fendleri*)	21, 16, 62 (Chen *et al*., 2011)	10 : 1 (Bhandari & Bates, 2021)	
Pennycress (*Thlaspi arvense*)	16, 30, 50 (Jarvis *et al*., 2021)		PDCT (−32%, 0) (Jarvis *et al*., 2021)

^1^ Distinction between TAG biosynthesis from predominantly *de novo* DAG or having a substantial presence of PC‐derived DAG is based on lipid flux results with isotopically labeled glycerol (Column 3), and/or genetic knockout/knockdown of PC‐derived DAG producing enzymes leading to a change in oil content or a reduction in polyunsaturated fatty acid content (Column 4). The initial relative amount of TAG synthesis from PC‐derived DAG or *de novo* DAG varies within each species and is estimated from *in vivo* glycerol labeling publications. Due to different experimental conditions, developmental stages, and labeling time points, the PC : TAG labeling ratios should not be quantitatively compared between species, and are meant to essentially indicate low vs high flux through PC‐derived DAG. The percent FA composition may not add up to 100 due to exclusion of minor FAs or rounding. *De novo* FAs are those not further modified (elongation, desaturation, etc.) from that of *de novo* synthesis in the plastid. PDCT, phosphatidylcholine:diacylglycerol cholinephosphotransferase; PLD, phospholipase D; NPC6, nonspecific phospholipase C6; n.d., not determined; VL, very low; VLCFA, very‐long‐chain FAs; WT, wild‐type.

Generally, *de novo* synthesized 18:1 is incorporated into PC for modification through two primary mechanisms: (1) the acyl editing cycle, acyl‐CoA:lyso‐PC acyltransferase (LPCAT) transfers 18:1 from 18:1‐CoA to mostly the *sn*‐2 position of lyso‐PC, producing PC. The initial lyso‐PC may be produced from phospholipase A_2_ (PLA_2_) or reverse LPCAT activity on PC (Bates *et al*., 2012; Lager *et al*., 2015); (2) PC synthesis by CDP‐choline:DAG cholinephosphotransferase (CPT) or PC:DAG cholinephosphotransferase (PDCT; Vogel and Browse, 1996; Lu *et al*., 2009; Liu *et al*., 2015). Within PC, 18:1 can be desaturated by FAD2 and FAD3 to produce linoleic acid and linolenic PUFAs in all plants, or modified by species‐specific enzymes, such as hydroxylases, acetylenases, and oxygenases to generate various UFAs (Shanklin *et al*., 2009; Ohlrogge *et al*., 2018). These PC‐modified FAs are transferred to TAG through multiple pathways/mechanisms (i–iv below):

- Acyl editing and *de novo* DAG‐based TAG synthesis: the acyl editing cycle allows FAs modified in PC to reenter the acyl‐CoA pool either directly from reverse LPCAT or activation of hydrolyzed free FAs by LACS; thus, the acyl‐CoA pool contains a mixture of newly synthesized and modified FAs that can be utilized by Kennedy pathway enzymes as described above (Fig. 2b). For example, castor accumulates ricinoleic acid in TAG via hydroxylation of PC‐18:1 and subsequent acyl editing and *de novo* TAG synthesis (Bafor *et al*., 1991; Tables 1, 2).
- PC‐derived DAG‐based TAG synthesis: DAG for TAG synthesis can be derived from PC by various catabolic reactions including the reverse action of CPT, PDCT, hydrolysis of a phosphocholine group by a phospholipase C (PLC), or the activities of phospholipase D (PLD) to produce PA with subsequent hydrolysis to DAG by PAP (Bates, 2016). Because PC is the site for FA modification, PC‐derived DAG can contain more modified FAs than *de novo* DAG and provides an efficient route to accumulate modified FAs, such as PUFA (that are abundant in PC) into TAG (Fig. 2c). Table 2 provides evidence of various species that utilize PC‐derived DAG for TAG biosynthesis as elucidated by *in vivo* isotopic tracing or characterization of mutants.
- Acyl‐CoA‐independent TAG synthesis: Phospholipid:DAG acyltransferase (PDAT) directly transfers FAs from *sn*‐2 PC to the *sn*‐3 position of *de novo* DAG or PC‐derived DAG, forming TAG and lyso‐PC (thus both anabolic and catabolic). PDAT activity combined with LPCAT activity (to resynthesize PC) can lead to a channeling of nascent 18:1 exported from the plastid into PC for modification and subsequent incorporation into TAG (Fig. 2b,c; Dahlqvist *et al*., 2000; Pan *et al*., 2013; Sah *et al*., 2024).
- TAG remodeling: TAG is not a metabolic endpoint, and during the oil accumulation stage of seed development, some plants actively remodel TAG by removing FAs from TAG for further modification and utilize the co‐produced DAG for the synthesis of new TAG molecular species; thus, the oil composition is optimized by an anabolic‐catabolic cycle. TAG remodeling requires the combined action of an acyl selective TAG lipase and selective acyltransferases (DGAT or PDAT) that can utilize the *sn*‐1,2‐DAG and *sn*‐2,3‐DAG enantiomers produced by the lipase (Fig. 2d; Bhandari & Bates, 2021; Parchuri *et al*., 2024). For example, *Physaria fendleri*, which accumulates HFAs, was shown to use a PC‐derived DAG pathway to first produce TAG containing one *sn*‐3 HFA, which is subsequently remodeled to a TAG molecular species containing HFA at both *sn*‐1/3 (Bhandari & Bates, 2021; Parchuri *et al*., 2024). Thus, TAG remodeling allows UFAs to accumulate in TAG, thereby decreasing their incompatible presence in membrane lipid intermediates. A similar mechanism has also been reported in the moss *Ceratodon purpureus*, where acetylenic 18:2 FAs are removed from TAG, desaturated, and reincorporated into new TAG molecules (Beutelmann & Stymne, 1995).

Although the core enzymes involved in TAG biosynthesis are broadly conserved among oilseed plants, the regulation of acyl flux through the lipid metabolic network into TAG remains poorly understood. The mere presence of key enzymes often fails to predict actual flux patterns or the resulting FA composition of TAG, as the same enzymes can be involved in multiple different branches of the lipid metabolic network (Fig. 2; Table 2). Fluxes are tightly regulated by a combination of factors, including enzyme substrate specificity, the spatial compartmentalization of enzymes and lipid intermediates, lipid transport processes, and developmental stage‐specific cues (Bates, 2016). These interconnected regulatory layers create substantial bottlenecks in engineering plants to produce UFAs, as the balance between lipid synthesis and degradation pathways differs between native and engineered systems. Moreover, the specific determinants that control acyl flux by kinetic, spatial, or transcriptional means remain largely undefined. Unraveling the mechanisms that determine pathway flux is essential for the rational design of bioengineered crops to accumulate target FAs efficiently without compromising seed oil yield or viability.

### 1. Engineering enhanced TAG assembly with unusual FA

**Fig. 3:**
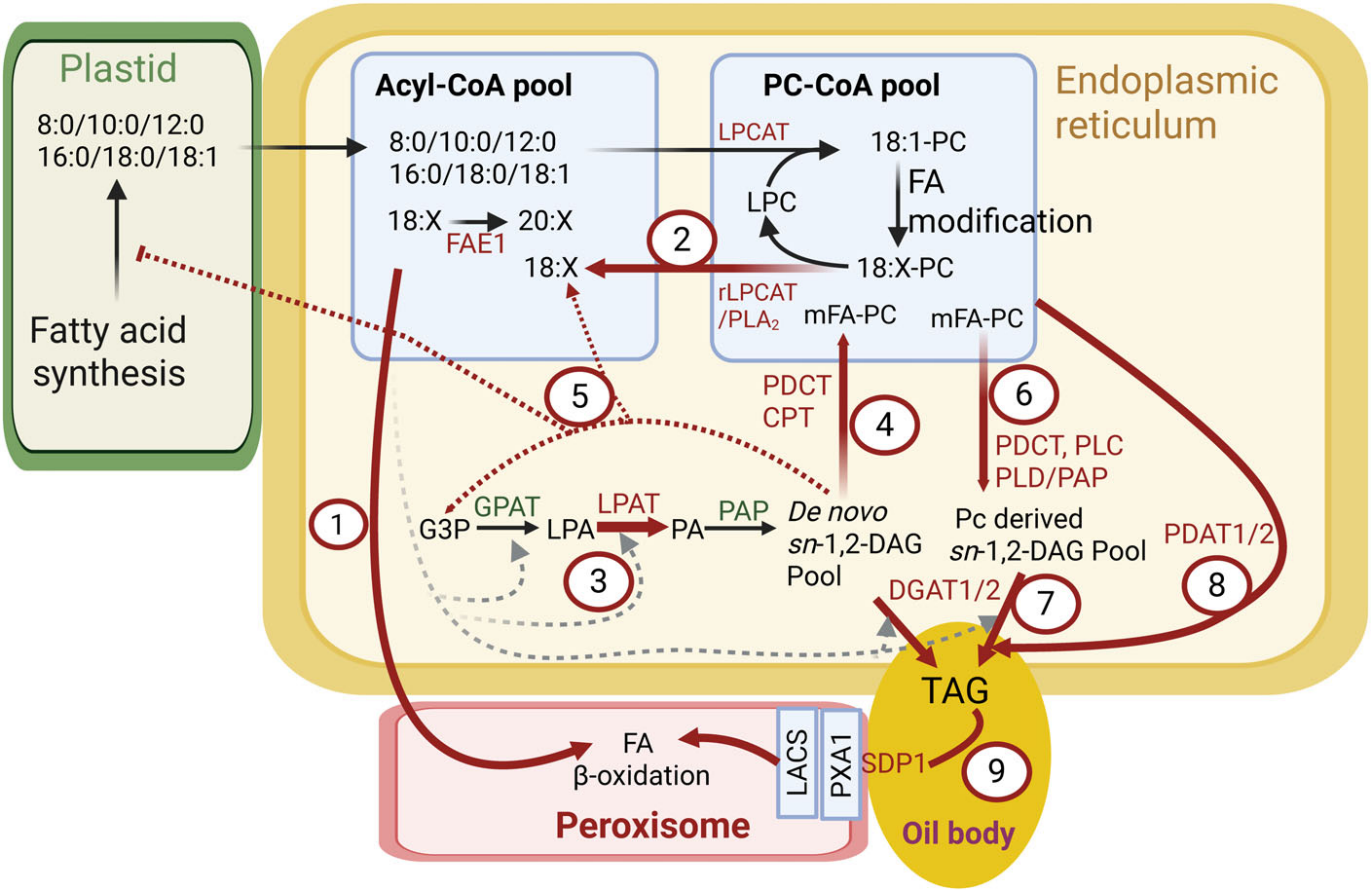
Key bottlenecks for engineering unusual fatty acids (UFAs) in *Arabidopsis* and other oilseed plants. For full names of genes and substrates, refer to the legend of Fig. 2. Thick red arrows indicate major bottlenecks and enzymes that are targeted for engineering. General cellular locations are indicated but luminal vs cytosolic facing reactions are not specified. Bottleneck 1: inefficient utilization of UFAs from the Acyl‐CoA pool triggers β‐oxidation of UFAs. Bottleneck 2: inefficient removal of modified fatty acids from phosphatidylcholine (PC) by acyl editing enzymes. Bottleneck 3: LPAT selects common FA substrates over UFA substrates. Bottleneck 4: inefficient production of UFA‐PC from UFA‐DAG. Bottleneck 5: futile cycle of *de novo* UFA‐DAG synthesis and degradation leading to feedback inhibition of FA synthesis by an unknown mechanism (red dashed arrows). Bottleneck 6: inefficient production of PC‐derived UFA‐DAG from UFA‐PC. Bottleneck 7: host DGAT1/2 selects against UFA substrates and UFA‐CoA. Bottleneck 8: host PDAT1/2 selects against UFA substrates. Bottleneck 9: degradation of UFA‐TAG by lipases and channeling of released UFAs for β‐oxidation in peroxisomes. Gray dashed lines are Kennedy pathway acyltransferase reactions. See Supporting Information Table S1 for engineering strategies used to overcome these bottlenecks. Substrate abbreviation: DAG, diacylglycerol; G3P, glycerol‐3‐phosphate; LPA, lyso‐phosphatidic acid; LPC, lysophosphatidylcholine; mFA, PC‐modified FA; PA, phosphatidic acid; PC, phosphatidylcholine; TAG, Triacylglycerol. Enzyme abbreviations: DGAT, acyl‐CoA:DAG acyltransferase; CPT, CDP‐choline:DAG cholinephosphotransferase; GPAT, acyl‐CoA:G3P acyltransferase; LPAT, acyl‐CoA:LPA acyltransferase; LPCAT, acyl‐CoA:LPC acyltransferase; PAP, PA phosphatase; PDAT, phospholipid:DAG acyltransferase; PDCT, PC:DAG cholinephosphotransferase; PLA_2_, phospholipase A_2_; PLC, phospholipase C; PLD/PAP, phospholipase D/PAP. Created in BioRender. Bates, P. (2025) https://BioRender.com/5cb08ml.

The production of UFAs (such as hydroxy, epoxy, and MCFAs) in high‐yielding oilseed crops holds immense promise for generating high‐value oils tailored for industrial and biofuel applications. Recent efforts to transfer UFA biosynthetic enzymes into genetically tractable Brassicaceae species (e.g. *Arabidopsis*, *Camelina*, and pennycress) have achieved only modest success, due to incompatibilities with their native lipid metabolic pathways (Fig. 3; Table S1). For example, introducing the *Ricinus communis* FA hydroxylase (*RcFAH12*) or *Cuphea* FatB genes (for MCFAs) into these species resulted in significantly lower accumulation of target UFAs (*c*. 9–17% for ricinoleic acid and 10–30% for MCFAs) compared with the *c*. 90% UFA in castor and *Cuphea* oils, respectively (Smith *et al*., 2003; Lu *et al*., 2006; Kim *et al*., 2015a,b; Iskandarov *et al*., 2017). More critically, these engineered Brassicaceae lines often exhibit a dramatic reduction in total seed oil content and defects in seed development, including size, weight, germination, and seedling vigor (Smith *et al*., 2003; Lu *et al*., 2006; Li *et al*., 2010; Bates *et al*., 2014; Kim *et al*., 2015a; Bansal *et al*., 2018; Yu *et al*., 2018; Esfahanian *et al*., 2021; Lunn *et al*., 2021). The primary bottleneck lies in the transgenic plants' biosynthetic capacity to efficiently channel modified FAs from their site of production on PC (in the case of HFA) or acyl‐CoA pool (in the case of MCFA) to their final storage in TAG. Key challenges include inefficient flux of modified FAs through the lipid metabolic network into TAG, substrate specificities of acyltransferases, differential DAG pool utilization, competing host lipid metabolism, and UFA‐induced catabolic reactions (Fig. 3; Table S1). In the following sections, we discuss key bottlenecks limiting the accumulation of specific UFAs in engineered plants relevant for biofuel and oleochemical applications.

### 2. Pathway bottlenecks in TAG biosynthesis limit UFA accumulation

Efforts to engineer UFAs into oilseed crops are often limited by metabolic incompatibilities between the native and host species, particularly regarding the flux of UFA intermediates through the lipid biosynthetic network into TAG. Many UFAs are incompatible with membrane lipid structure and function (e.g. hydroxy and MCFA). Thus, engineering UFAs into oilseed species which rely on the PC‐derived DAG pathway can trigger futile cycles of lipid synthesis and degradation or buildup of UFA lipid intermediates leading to feedback inhibition of FAS (Section II), ultimately reducing seed oil content and occasionally causing developmental abnormalities (Fig. 3; Table S1; Bates & Browse, 2011; Bates *et al*., 2014; Iskandarov *et al*., 2017). For example, *in vivo* isotopic tracing of lipid metabolism in HFA‐producing transgenic *Arabidopsis* lines revealed that the conversion of *de novo* HFA‐DAG to HFA‐PC is a significant bottleneck for efficient accumulation of HFA into TAG by the PC‐derived DAG pathway (Bates & Browse, 2011) and contributes to post‐translational downregulation of ACCase activity, reducing FAS and oil accumulation (Fig. 3; Bates *et al*., 2014). These results suggest that native *Arabidopsis* enzymes, such as PDCT and CPT, preferentially utilize non‐HFA molecular species, limiting the flux of HFA‐containing DAG into PC and subsequently into TAG (Bates & Browse, 2011). The expression of castor PDCT in *Arabidopsis* partially alleviated this constraint (Hu *et al*., 2012). However, the selectivity of castor PDCT for *sn*‐2 mono‐HFA‐DAG (rather than DAG containing two HFA) may continue to be a constraint within bioengineering (Demski *et al*., 2022).

Similarly, *Arabidopsis* engineered to produce CPFAs also suggest inefficient flux through the PC‐derived DAG pathway (Table S1). CPFAs produced via *E. coli* CPS (EcCPS) expression accumulated mainly in membrane lipids, with limited transfer to TAG. Co‐expression of *Sterculia foetida* PDCT partially alleviated this constraint, increasing CPFA accumulation in TAG by *c*. 50% compared with the EcCPS line alone (Yu *et al*., 2018, 2019). Although detailed metabolic flux studies are lacking, these observations demonstrate that CPFA flux through the PC‐derived DAG pathway is a potential bottleneck in engineered plants.

These examples highlight how differences in lipid flux through TAG biosynthetic mechanisms (e.g. Kennedy pathway vs PC‐derived DAG) between native and engineered plants present significant challenges for successful bioengineering. Emerging evidence from *Arabidopsis* suggests that lipid metabolism may be organized into enzyme–enzyme assemblies that mediate substrate channeling between them, a phenomenon known as a metabolon. It has been hypothesized that membrane lipid and TAG biosynthetic enzymes are spatially separated within the ER and PC acts as a dynamic carrier of DAG molecules between these two metabolons (Regmi *et al*., 2020; Bates, 2022; Bates & Shockey, 2025). If such metabolon‐like structures are indeed common in PC‐derived DAG pathway‐utilizing plants, they may impose additional constraints on engineering UFA‐enriched oils where the UFAs are not compatible with membrane lipid structure–function. To circumvent these constraints, one promising strategy involves assembling artificial metabolons by co‐expressing multiple enzymes from native UFA‐producing species in a coordinated manner, or as fusion proteins (Xu *et al*., 2020, 2023). However, this approach often requires extensive modification of host lipid pathways, including the elimination/suppression of endogenous enzymes that compete for substrates. Additionally, TAG biosynthesis is essential in pollen, thus requiring any replacement of endogenous TAG biosynthesis to be expressed with the correct spatiotemporal profile for all physiological functions of TAG, not only seed oil accumulation (McGuire *et al*., 2025a,b). Alternatively, inducing TAG remodeling as discovered in *Physaria fendleri* offers a compelling route (Bhandari & Bates, 2021; Parchuri *et al*., 2024). In this mechanism, initial TAG molecules are synthesized from PC‐derived DAG, followed by postsynthetic remodeling steps that selectively enrich TAG with UFAs that are otherwise incompatible with membrane lipid intermediates. Exploring and integrating such alternative pathways, including both metabolon formation and TAG remodeling, will be critical for overcoming flux limitations and achieving high‐efficiency, sustainable production of UFAs in commercial oilseed crops.

### 3. Substrate selectivity of acyltransferases control UFA flux into TAG in engineered oilseeds

Engineering the production of UFAs in conventional oilseed crops often begins with the expression of a single key UFA biosynthetic enzyme. However, this approach typically results in low UFA accumulation compared with native UFA‐producing species (Tables 1, S1). In transgenic plants, UFA‐CoAs can accumulate in the acyl‐CoA pool rather than being efficiently incorporated into TAGs, often triggering β‐oxidation and futile cycles of synthesis and degradation (Poirier *et al*., 1999; Larson *et al*., 2002; Moire *et al*., 2004; Fig. 3). A major limitation is the selectivity of host species Kennedy pathway enzymes (GPATs, LPATs, and DGATs) toward endogenous FAs rather than the engineered UFAs.

Biochemical studies in native UFA‐accumulating species have revealed that Kennedy pathway enzymes exhibit species‐specific substrate selectivity, which contributes to the distinct UFA‐enriched TAG profiles observed in these plants (Bafor *et al*., 1990, 1991; Jeppson *et al*., 2019; Lager *et al*., 2020; Chen *et al*., 2022; Parchuri *et al*., 2024). Furthermore, most plant genomes encode multiple isoforms of each Kennedy pathway enzyme – up to 10 GPATs, five LPATs, and at least three DGATs, with many isoforms displaying differential acyl donor and acyl acceptor substrate preferences (Shockey *et al*., 2016; Singer *et al*., 2016; Kim *et al*., 2020; Barroga & Nakamura, 2022; Zhang *et al*., 2022). For example, *Cuphea lanceolata* expresses distinct GPAT and LPAT isoforms that separately direct MCFAs and long‐chain FAs into different DAG pools. Its DGAT further enhances medium‐chain TAG biosynthesis by preferentially acylating di‐10:0 DAG with 10:0‐CoA (Bafor *et al*., 1990). Similarly, microsomal assays in castor have shown that GPAT and LPAT enzymes generate DAGs enriched in HFAs, which are then acylated by DGATs to form tri‐HFA TAGs (Bafor *et al*., 1991). Beyond acyl selectivity, recent findings also reveal that DGAT1 and DGAT2 differ in their enantiomeric preferences for DAG substrates in *Physaria*, with DGAT1 favoring *sn*‐1,2‐DAG and DGAT2 preferring *sn*‐2,3‐DAG. The *sn*‐2,3‐DAG is produced by partial TAG catabolism by a TAG lipase, and thus both activities are key for the accumulation of UFA through TAG remodeling (Parchuri *et al*., 2024). However, whether such enantiomeric specificities exist in other oilseed crops remains unknown.

Engineering UFA synthesis combined with the expression of UFA‐selective acyltransferases has been a successful approach to increase UFA content of seed oils and can also mitigate detrimental phenotypes, such as reduced total oil content due to inefficient utilization of UFA often associated with the expression of UFA biosynthetic genes alone (Table S1). These findings underscore the critical importance of selecting acyltransferases with suitable substrate specificities to facilitate efficient flux of UFAs into TAGs, yet information on the DAG molecular species and acyl‐CoA selectivity of most plant acyltransferases is limited and cannot yet be determined from protein sequence alone. Therefore, detailed biochemical characterization of Kennedy pathway isoforms, both in native UFA‐producing species and in heterologous systems, will be required to effectively direct UFAs into storage lipids in conventional oilseed crops.

### 4. Emerging role of PDAT in engineering: tuning membrane and storage lipid interfaces

PDATs represent a distinct class of acyltransferases that contribute to TAG biosynthesis in an acyl‐CoA independent manner (Dahlqvist *et al*., 2000; Ståhl *et al*., 2004; Sah *et al*., 2024). PDAT1 and PDAT2 from different species exhibit high selectivity for both acyl donors and DAG species, preferentially channeling modified FAs from PC into TAG (Table S1; Pan *et al*., 2013; Yuan *et al*., 2017; Lager *et al*., 2020; Parchuri *et al*., 2022). This links acyl editing with TAG assembly and is supported by LPCAT‐mediated PC regeneration, forming a metabolic cycle that facilitates continuous flux of FAs into PC for modification and modified FAs into storage lipids (Xu *et al*., 2012; Lunn *et al*., 2020). In plants, PDAT1 plays a secondary but critical role in seed oil production, especially under conditions where DGAT1 is impaired. While *Arabidopsis pdat1* mutants show no major oil phenotype, PDAT1 compensates for *c*. 80% of TAG biosynthesis in the *dgat1‐1* background, indicating its essential role when functional DGAT1 is absent (Zhang *et al*., 2009). Notably, *in vivo* flux analysis in the *dgat1‐1* mutant showed that AtPDAT1 accesses a distinct pool of PC‐derived DAG compared with AtDGAT1, suggesting that AtPDAT1 does not function as a direct substitute for AtDGAT1 in TAG biosynthesis (Regmi *et al*., 2020). A similar compensatory mechanism was observed in *Physaria fendleri*, where knockdown of *PfeDGAT1* or *PfeDGAT2* led to a significant upregulation of *PfePDAT1*, likely to maintain TAG synthesis. Notably, in the *PfeDGAT2* knockdown, *PfePDAT1* expression had a much stronger compensatory response than for *PfeDGAT1 knockdown*, suggesting that *PfePDAT1* utilizes the *sn*‐2,3 DAG generated during TAG remodeling similar to PfeDGAT2 (Parchuri *et al*., 2024). However, direct evidence for PDAT DAG enantiomer selectivity has yet to be demonstrated.

Overexpression of PDAT1 or PDAT2 enhanced UFAs in TAG and altered FA profiles in transgenic plants, although typically less than DGAT1 overexpression (Table S1). PDATs are also linked to membrane lipid remodeling and stress responses (Sah *et al*., 2024; Shomo *et al*., 2024), suggesting the primary role of PDATs may be in maintaining membrane homeostasis during stress or periods of high UFA synthesis rather than directly maximizing oil yield. In engineered plants producing UFAs, PDAT may help sequester membrane‐incompatible FA into TAG, mitigating membrane disruption. However, the extent to which PDAT, as opposed to DGAT, contributes to TAG accumulation under different physiological conditions remains unclear. Future work should dissect the individual roles and regulation of PDAT isoforms across tissues and species. Mapping their interactions with DAG and PC pools, and integrating them into broader lipid networks will improve their utility in oilseed engineering.

### 5. Differential DAG pool utilization and substrate channeling influence TAG engineering

Accumulating evidence indicates that substrate preferences of acyltransferases are not dictated by enzymatic selectivity alone, but are also influenced by the *in vivo* biochemical context and lipid metabolic organization of the host species (Regmi *et al*., 2020; Bates & Shockey, 2025). In particular, the source and molecular identity of DAG pools used for TAG synthesis appear to be species‐ and isoform‐specific, contributing to variations in TAG composition and UFA accumulation (Regmi *et al*., 2020). For example, RcDGAT2 in *Ricinus communis* preferentially uses *de novo* DAG from the Kennedy pathway (Bafor *et al*., 1991; Burgal *et al*., 2008), while AtDGAT1 and PfeDGAT1 utilize PC‐derived DAG (Regmi *et al*., 2020; Parchuri *et al*., 2024). In contrast, PfeDGAT2 prefers *sn*‐2,3‐DAG generated from TAG remodeling (Fig. 2d; Parchuri *et al*., 2024). However, transgenic expression studies suggest that these DAG preferences are not always retained when DGATs are heterologously expressed. When RcDGAT2 is expressed in *Arabidopsis dgat1‐1*, it fails to use *de novo* DAG or initially produced PC‐derived DAG; instead, it utilizes a more slowly turned‐over PC‐derived DAG pool that is also used by AtPDAT1 (Regmi *et al*., 2020), suggesting that substrate utilization is influenced by integration into the host plants' lipid metabolic network. Furthermore, it is likely that an introduced acyltransferase may require interaction with host proteins to successfully integrate into a lipid biosynthetic metabolon (McGuire *et al*., 2025b). Such findings emphasize the importance of considering DAG pool availability in an engineered host and compatibility with expressed UFA‐selective acyltransferases in metabolic engineering strategies. Therefore, effective engineering may require not only co‐expression of UFA and TAG biosynthetic enzymes but may also require remodeling of endogenous lipid metabolic pathways of the host or generation of artificial metabolons through protein fusions.

### 6. Targeted engineering and suppression of endogenous pathways enhance UFA flux into TAG

Efforts to engineer the accumulation of UFAs in seed oils are frequently hindered by competition between transgene‐derived enzymes and the corresponding endogenous enzymes. Recent studies demonstrate the effectiveness of targeted suppression of endogenous acyltransferases in improving engineered oil profiles. For example, in *Camelina*, both mutation of *fae1* and suppression of endogenous *DGAT1* significantly enhanced acetyl‐TAG (TAG with acetate group at *sn*‐3 position) accumulation when *Euonymus fortunei* diacylglycerol acetyltransferase (*EfDAcT; an enzyme that esterifies the acetate group to the DAG to form TAG*) was expressed (Alkotami *et al*., 2024). This result underscores the role of endogenous DGAT activity and FA selectivity that may hinder bioengineering success. In a more comprehensive demonstration, McGuire *et al*. (2025b) successfully replaced the activity of both endogenous *DGAT1* and *PDAT1* in *Arabidopsis* with an exogenous copy of DGAT1s from *Arabidopsis*, *Camelina*, and *Physaria fendleri* (despite the *dgat1/pdat1* male gametophyte lethality (Zhang *et al*., 2009)) by effectively capturing DGAT1 spatial and temporal expression patterns (McGuire *et al*., 2025b). Proper expression of *AtDGAT1* in pollen is controlled by both the promoter and the *AtDGAT1* first intron, which are crucial to pollen development and fertility. This study not only demonstrates the feasibility of functionally replacing endogenous acyltransferases with heterologous copies but also underscores the critical role of preserving native *cis*‐regulatory architecture to achieve proper spatial and temporal expression. Future work should determine whether similar regulatory elements are required in other species, particularly in polyploids, such as *Brassica napus*, where duplicated DGAT homologs often display tissue‐specific expression patterns in pollen or seeds (Yang *et al*., 2023). Together, these findings highlight that metabolic engineering of seed oils must go beyond simple transgene overexpression and consider the complex interplay between introduced and native pathways. Reducing or replacing competing endogenous activities while maintaining appropriate developmental regulation represents a powerful strategy to control FA compositions in TAGs (McGuire *et al*., 2025a,b).

## V. Modulation of lipid catabolism for enhanced oil bioengineering

**Fig. 4:**
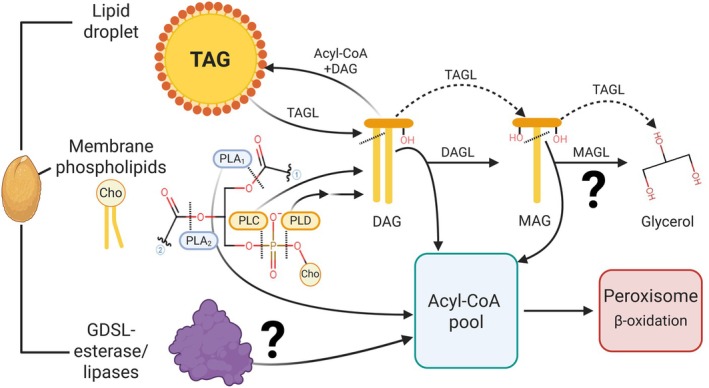
Contribution of phospholipid and neutral lipid lipases in tuning distinct lipid pools in seeds. All membrane glycerophospholipids and neutral lipids can be targets of lipase activity. The most abundant lipids during seed development are TAGs residing in lipid droplets and membrane phospholipids. Cho indicates a choline headgroup on phosphatidylcholine. The release of fatty acids by different lipases contributes to either DAG, MAG, or acyl‐CoA pools. Dashed lines in each molecule indicate the ester bond hydrolyzed by each lipase. Dashed lines with arrowheads denote that some TAGLs can perform complete hydrolysis to produce glycerol while others cleave the *sn‐*1,3 positions to produce MAG. Question marks indicate outstanding unknowns of the relative contribution of these lipases during seed oil deposition. Presently, the substrates of most GDSL‐lipases/esterases remain undefined. The acyl‐CoA produced by lipases can be further catabolized in the peroxisome via β‐oxidation. Enzyme abbreviations: PLA1/2, phospholipase A1/2; PLC, phospholipase C; PLD, phospholipase D; TAGL, triacylglycerol lipase; DAGL, diacylglycerol lipase; MAGL, monoacylglycerol lipase. Created in BioRender. Bates, P. (2025) https://BioRender.com/noeoxc2.

During seed germination and establishment, storage oil mobilization by TAG lipases is essential (Eastmond, 2006; Kim *et al*., 2011; Ding *et al*., 2019). Various glycerolipid lipases can also be involved in signaling and plant development, which has been reviewed (Kelly & Feussner, 2016; Wang *et al*., 2019). Most oil bioengineering research has focused on the anabolic reactions of the Kennedy pathway during seed‐filling and oil deposition, and relatively little is known about catabolic lipase activities during oil accumulation (Muñoz *et al*., 2019; Zhou *et al*., 2020; Lunn *et al*., 2021). Glycerolipid lipases can modulate the composition of metabolite pools utilized during oil synthesis (e.g. PC, DAG, and TAG) and can be bottlenecks for engineering plant oils (Fig. 4).

### 1. Illuminating the roles of lipases acting on neutral lipid pools

The enzymatic actions of neutral lipid lipases acting on monoacylglycerol, diacylglycerol, and triacylglycerol (MAGL, DAGL, and TAGL) release a free FA along with a glycerol, 2‐monoacylglycerol, or DAG (*sn‐*1,2 and *sn‐*2,3), respectively. These products can be substrates for anabolic reactions to generate new lipid molecular species or be further catabolized to supply other parts of metabolism.

The action of TAG lipases during seed oil accumulation can produce two outcomes: complete TAG turnover and FA β‐oxidation resulting in reduced oil content; or TAG remodeling (Figs 2d, 4). The major TAG lipase Sugar‐Dependent 1 (SDP1; Eastmond, 2006; Kelly *et al*., 2011) expressed during seed maturation cleaves FA at the *sn*‐1,3 positions and leads to *c*. 10% reduction in total seed oil in many species. The β‐oxidation of FAs released by SDP1, rather than reutilization, is likely promoted by the localization of SDP1 at the interface of lipid droplets (LDs) and the peroxisome (Thazar‐Poulot *et al*., 2015). The targeted suppression of *SDP1* increases oil content and seed yields in agronomically important crops, such as *Glycine max* (soybean; Kanai *et al*., 2019; Aznar‐Moreno *et al*., 2022) and *Brassica napus* (Kelly *et al*., 2013) without a change in FA composition. However, suppression of *SDP1* in *Physaria fendleri* led to both increased oil content and HFA amount, indicating that TAG degradation by SDP1 can be FA selective and can be targeted to control oil composition (Azeez *et al*., 2022). Opposite to the effect of SDP1 is the ER‐oil body localized TAGL of *Physaria fendleri* (*PfeTAGL1*) involved in TAG remodeling where the FA released from *sn*‐1 TAG re‐enters lipid metabolism for further modification, and a different FA is added to the *sn*‐2,3‐DAG by PfeDGAT2 to generate new TAG molecular species (Parchuri *et al*., 2024). To date, it is unknown whether other oilseed plants control TAG FA composition by TAG remodeling; however, the large number of uncharacterized genes annotated as TAG lipases (Horn *et al*., 2016) indicates their roles in TAG turnover or remodeling remain unexplored.

The interplay between catabolic and anabolic reactions involved in ‘TAG remodeling’ may go beyond the TAG lipase/DGAT mechanism characterized in *Physaria fendleri* and involve further ‘fine‐tuning’ of DAG and MAG pools available to acyltransferases. For example, overexpression studies of peanut (*Arachis hypogaea*) MAGL in *Arabidopsis* decreased oil content and led to compositional changes with higher PUFA (18:2) and decreased 20:1 content (Zhan *et al*., 2023). Furthermore, AtMAGL8 is associated with oil bodies during germination (Kim *et al*., 2016), and likely acts after the removal of TAG *sn*‐1,3 FA by TAG lipases. Although it remains unclear whether MAGLs influence the MAG or acyl‐CoA pools available for DAG/TAG synthesis (or TAG remodeling) during oil accumulation in developing seeds, results in *Gossypium hirsutum* (cotton) identified *MAGL3* and *MAGL6* as candidate genes that are downregulated during seed development in accessions containing > 2% increased seed oil (Zhou *et al*., 2024). Analogously, a DAGL homologous to the human *ADIPOSE TRIGLYCERIDE LIPASE‐LIKE* (*ATGLL*) balances the prokaryotic (plastidial) and eukaryotic (ER) glycerolipid pools to contribute MUFA or PUFAs to acyltransferases in either organelle. In the vein of engineering, *ATGLL* overexpression increased leaf TAG content fourfold, while T‐DNA knockout and RNAi‐mediated suppression lines decreased total oil (Yu *et al*., 2023). Thus, revealing that FA alterations in membranes of either organelle influence TAG production. As with TAG lipases, the large number of genes annotated as MAGL or DAGL likely originated from duplication events allowing for differential expression during seed development, which suggests that various neo‐functionalized roles of these enzymes can be further characterized and exploited to control seed oil content.

### 2. Phospholipase activity facilitates oil production and modifies oil content

#### Production of DAG precursors: phospholipid turnover mechanisms modifying seed oil content

As stated above, multiple mechanisms produce DAG for TAG synthesis (see Section IV). For instance, the nonspecific phospholipase C (NPC) class can contribute to the composition of the PC‐derived DAG pool. A pair of NPCs, NPC2 and NPC6, supply DAGs for acylation in developing pollen tubes (Bose *et al*., 2021). Overexpression studies in *Arabidopsis* (increased oil 6.0–8.1%) and *Camelina* (increased oil 6.3%) demonstrate that NPC activity provides DAG during oil deposition.

Another phospholipase, PLD, yields a PA molecule that can be dephosphorylated into DAG for incorporation into TAG. Many PLD isoforms exist, and several are characterized in effecting seed oil production. For example, suppression of *PLDα* in soybean reduced PUFA in the seed oil while maintaining oil yield (Lee *et al*., 2011). While in *Camelina*, overexpression of PLDζ increased total oil content by 3% (Yang *et al*., 2017). Additionally, one proposed side effect of PA is that it activates DGAT1 for enhanced TAG synthesis (Caldo *et al*., 2018). Collectively, many phospholipases contribute DAG molecular species and, as such, are promising candidates for adjusting DAG pool compositions for target oil production.

#### Reutilization of FA released by lipases requires production of acyl‐CoA precursors

How can UFA‐accumulating species readily remove membrane‐incompatible FAs from the site of synthesis in the membrane and channel them toward TAG production? One known mechanism is that catalyzed by PLA_2_ that hydrolyzes FAs from the *sn‐*2 position of PC. An excellent example of a specific PLA_2_ is that from castor, which efficiently removes HFA from PC; this HFA is then re‐activated and then readily incorporated into TAG by RcDGAT2 (Burgal *et al*., 2008; Li *et al*., 2010). However, the knowledge surrounding PLA_2_ and specialized FAS does not always translate into a heterologous context. In HFA‐producing *Arabidopsis*, the expression of castor *PLA*_*2*_ removes HFA from PC; however, little of the HFA is incorporated into TAG (Bayon *et al*., 2015). This inefficiency of reincorporation of HFA into TAG is likely due to the incompatibility of HFA with the endogenous LACS enzymes. Across the plant kingdom, many PLA isoforms exist, with PLAIIIδ recognized as important in seed oil accumulation. In camelina, seed‐specific expression produced 9% higher seed oil (Liu *et al*., 2015). Since PLA activity releases nonesterified FAs from the PC pool, these FAs must rely on being activated once more by LACS and then shuttled by an ACBP or directly esterified to a recipient molecule (e.g. MAG, LPA, LPC, and DAG) by a nearby acyltransferase. Otherwise, they are likely destined for degradation through β‐oxidation.

### 3. Exploring knowns and unknowns of GDSL‐motif enzymes

Over 300 genes are putatively annotated as ‘lipases’, but experimental evidence confirming their *in planta* roles is available in only a few cases (Li‐Beisson *et al*., 2013; McGlew *et al*., 2015). Furthermore, the true enzymatic identity of many lipases awaits empirical investigation, and many may have bifunctional activities that can act as a ‘lipase’, ‘acylhydrolase’, or ‘esterase’. Simple gene annotation is insufficient for describing these often multifaceted proteins. In particular, the evolutionarily conserved multigene GDSL esterase/lipase protein (GELP) family composed of 105 members (in *Arabidopsis*) is mostly classified solely from protein domain conservation (Lai *et al*., 2017). Yet, their roles in lipid breakdown or accumulation remain unclear.

In *Brassica napus*, targeted suppression of *GDSL1* increased oil content in the seed but caused germination defects, while constitutive overexpression reduced total oil content, indicating that this gene does in fact act as a lipase (Ding *et al*., 2019). One successful engineering case is the GELP known as seed FA reducer (*SFAR*) where the *sfar1‐3/sfar3‐1* double mutant increases seed FA content by 31.8% (Chen *et al*., 2012). Additionally, the *SFAR* knockout phenotype appears to be conserved, as CRISPR‐Cas‐mediated removal of *SFAR4* in *Brassica napus* increased seed LD area and oil content with no detrimental growth characteristics (Karunarathna *et al*., 2020). Due to the diversity of this gene family, many other uncharacterized GELPs have been interpreted to be important in maize accumulating > 20% oil by kernel weight when compared to conventional sweet corn (Luo *et al*., 2023) and also in high oil accessions of *Sesamum indicum* (Sesame; Dutta *et al*., 2022). Thus, this gene family should be carefully dissected to identify candidates for biotechnological applications. Although difficult to define their exact physiological roles, GELPs show promise as being important in lipid metabolism, embryo development, and seed oil accumulation.

## VI. Conclusion and future outlook

Substantial progress has been made in elucidating the enzymatic framework underlying FAS and oil accumulation in plants. However, engineering oilseed crops to accumulate high levels of UFAs continues to be constrained by metabolic incompatibilities, pathway bottlenecks, and complex regulatory networks. This review highlights that effective lipid engineering requires a deep understanding of acyl flux control, shaped by substrate selectivity of enzymes, differential substrate pool utilization, dynamic lipid remodeling, interplay between anabolic and catabolic lipid pathways, and cellular organization of lipid metabolism. A recurring challenge is the poor integration of UFA biosynthetic enzymes into host metabolic networks, leading to inefficient flux, accumulation of undesirable intermediates, or penalties in seed development and yield (see, additional insights in Box 1 and outstanding questions in Box 2).

Box 1 **Insights on industrial oil bioengineering**

1. Unusual fatty acid (UFA) incompatibility with host species lipid metabolism can cause feedback inhibition or futile cycles of synthesis and degradation.
2. Host species that utilize a phosphatidylcholine (PC)‐derived diacylglycerol (DAG) precursor to triacylglycerol (TAG) may limit the flux of membrane‐incompatible FAs from *de novo* DAG into PC, and thus into TAG.
3. Combining UFA synthesis with TAG assembly enzymes selective for UFA can increase their levels while limiting adverse effects of UFA production in host species.
4. A high proportion of UFA in engineered seed oils likely requires reducing competition from endogenous oil biosynthetic pathways.
5. Artificial metabolons utilizing fusion proteins may increase efficiency of UFA production and reduce competition from endogenous pathways.
6. TAG biosynthesis is essential in pollen, thus replacement of endogenous oil biosynthetic pathways with UFA‐selective pathways will require the correct expression context for both seed and pollen oil production.

Box 2 **Outstanding questions**

1. How can carbon and energy flux be dynamically redirected to fatty acid synthesis without compromising seed protein or carbohydrate reserves?
2. What are the precise molecular mechanisms regulating heteromeric ACCase activity, and how can they be leveraged to enhance fatty acid synthesis in a tissue‐specific manner?
3. To what extent can thioesterase specificity and expression timing be tuned to support stable accumulation of medium or unusual‐chain fatty acids in seeds?
4. What defines the functional compatibility of acyl‐CoA synthetases and acyl‐CoA binding proteins with unusual fatty acids in engineered systems?
5. How do endogenous lipid metabolic networks reshape flux in engineered plants expressing unusual fatty acid (UFA) biosynthetic pathways?
6. Can triacylglycerol (TAG) remodeling be leveraged to enrich TAG with unusual fatty acids?
7. Can metabolon formation be engineered or exploited to enhance substrate channeling and minimize metabolic leakage?
8. How are distinct diacylglycerol (DAG) pools organized, accessed, and regulated within the endoplasmic reticulum (ER), and how do they shape UFA incorporation into TAG?
9. What are the unknown lipases that act on DAG, monoacylglycerol (MAG), and TAG pools during seed development, and how do they influence oil content and composition?
10. What is the contribution of phospholipase‐mediated DAG and FA release to acyl flux in seed oil biosynthesis?

Ultimately, a holistic synthesis of synthetic biology and systems biology principles, including gene identification, cell biology, and biochemical characterization, metabolomics, fluxomics, and reduced competition from host metabolic pathways, will be essential for overcoming current limitations and building the next generation of designer oilseed crops tailored for food, fuel, and industrial applications. However, the connection between proof‐of‐concept engineering in model species and achieving stable, high‐yield performance in the field is rarely direct (Uauy *et al*., 2025). While model species are valuable for elucidating gene function and evaluating gene candidates, identifying effective engineering strategies requires understanding how variable field conditions affect final seed oil yield and composition. Translational success should therefore be defined not only by enhanced accumulation of desired FAs under controlled conditions but also by sustained oil yield, composition, and seed vigor in the field. Close coordination between predictive metabolic redesign, controlled‐environment studies, and multi‐location field trials will be critical for refining engineering strategies and anticipating environmental influences. Bridging this lab‐to‐field continuum will ultimately determine the true success of oilseed crop engineering in the future.

## Competing interests

None declared.

## Disclaimer

The New Phytologist Foundation remains neutral with regard to jurisdictional claims in maps and in any institutional affiliations.

## Supporting information


**Table S1** Genetic engineering strategies for unusual fatty acid production in oilseeds and insights from the results.
Please note: Wiley is not responsible for the content or functionality of any Supporting Information supplied by the authors. Any queries (other than missing material) should be directed to the *New Phytologist* Central Office.
