# Supplementary material for: Understanding the dynamic nature of plant lipid anabolic and catabolic metabolism is key to sustainable oilseed engineering
Source: New Phytol. 2025 Dec 24;249(5):2196–214. doi: 10.1111/nph.70849 (PMC12873504; doi:10.1111/nph.70849)
Supplement: Supplementary file 1 — Table S1 Genetic engineering strategies for unusual fatty acid production in oilseeds and insights from the results. Please note: Wiley is not responsible for the content or functionality of any Supporting Information supplied by the authors. Any queries (other than missing material) should be directed to the New Phytologist Central Office. [file NPH-249-2196-s001.pdf]

New Phytologist Supporting Information

Article title: Understanding the dynamic nature of plant lipid anabolic and catabolic metabolism is key to sustainable oilseed engineering

Authors: Prasad Parchuri, Sean T. McGuire, Matthew G. Garneau, Niña Alyssa M. Barroga, Philip D. Bates*

Article acceptance date: 18 November 2025

Supplementary Table S1: Genetic engineering strategies for UFA production in oilseeds and insights from the results.

Host plant	Transgene and Native species	UFA levels (% of total FAs); Seed oil content (%)	Bottlenecks/Key insight from engineering	References that supports the insights
Hydroxy fatty acid engineering				
Arabidopsis WT	-	0% HFA; ~35-37% seed oil	No unusual fatty acid production	(Li-Beisson *et al.*, 2013)
Arabidopsis WT, *fad2, fad2/fae1, fad3, fad3/fae1*	FA hydroxylase (FAH12) from *R. communis*	~ 9-19% HFA; ~20-23% seed oil	Low HFA accumulation due to limited flux of HFA into TAG via lipid metabolic network.	(Broun & Somerville, 1997; Smith *et al.*, 2003)
Arabidopsis fae1 mutant	FA hydroxylase (FAH12) from *R. communis*	~17% HFA; ~ 23% seed oil	Low HFA-TAG formation due to inefficient HFA-DAG to PC flux, futile HFA-DAG cycling, PDCT substrate bias, and feedback inhibition on ACCase	(Lu *et al.*, 2006; Bates & Browse, 2011; Bates *et al.*, 2014; Demski *et al.*, 2022)
	FAH12 and PDCT from *R. communis*	~23% HFA; 5.4 μg/seed (4.4 μg/seed in CL37 and 7.5 μg/seed in WT)	HFA levels were increased, but low seed oil phenotype was not recovered completely due to lack of preference of RcPDCT for *sn*-1 mono-HFA-DAG (major species in CL37).	(Hu *et al.*, 2012; Demski *et al.*, 2022)
	FAH12 and DGAT2 from *R. communis*	~27% HFA; ~32% seed oil	RcDGAT2 improved HFA-CoA incorporation into TAG, but seed oil recovery was limited due to differential DAG pool utilization in Arabidopsis.	(Burgal *et al.*, 2008; Regmi *et al.*, 2020)
	FAH12 and PDAT1 from *R. communis*	~25% HFA; ~26% Seed oil	RcPDAT1 promoted HFA transfer from PC to TAG, but failed to fully restore seed oil, possibly due to secondary role of PDAT1 in TAG synthesis and access to distinct DAG pools in Arabidopsis.	(van Erp *et al.*, 2011)
	FAH12, GPAT9, LPAT and DGAT2 from *R. communis*	~ 27% HFA; ~30% seed oil	Introducing three Kennedy pathway enzymes from castor was marginally better to RcDGAT2 alone due to inherent differences in TAG biosynthetic pathways and DAG pool compartmentalization between Arabidopsis and castor.	(Shockey *et al.*, 2019)

	FAH12, GPAT9, LPAT and PDAT1 from *R. communis*	~34% HFA;<br>~35 % seed oil	Efficient di-HFA-DAG synthesis by GPAT9/LPAT and transfer HFA from *sn*-2 PC to diHFA-DAG by PDAT1 enhanced HFA content. Removal of HFA from PC by PDAT1 lead to relief of ACCase inhibition, improved total FA synthesis and seed oil levels.	(Lunn *et al.*, 2019, 2022)
Camelina sativa	FAH12 from *R. communis*<br><br>RcFAH12 and KSC from *Lesquerella fendleri*	~15% HFA;<br>~145 μg/seed (~185 μg/seed WT)<br><br>20% HFA;<br>~175 μg/seed	Low HFA accumulation due to limited flux of HFA into TAG via lipid metabolic network. Introduction of KCS3 from Physaria increased HFA and recovered total seed oil content due to efficient channeling of 20 carbon HFA from acyl-COA pool and net movement of HFA out of PC.	(Snapp *et al.*, 2014)
Medium chain Fatty acid engineering				
Brassica napus WT	-	0% MCFA:<br>ND	No MCFA production	(Dehesh *et al.*, 1996; Voelker *et al.*, 1996; Poirier *et al.*, 1999; Larson *et al.*, 2002)
	FATB1 from *U. californica*<br><br>*FATB2 from C. hookeriana*	~50% MCFA (12:0); ND<br><br>~40% of MCFA (8:0, 10:0, 12:0); ND	High MCFA accumulation, but lower than native *Cuphea*. MCFA-CoA buildup leads to β-oxidation and futile synthesis-degradation cycles.	
	FATB1 from *U. californica* and LPAT from *Cocos nucifera*	~67% 12:0; ND	CnLPAT promotes MCFA incorporation at sn-2 of TAG. However, limited further gain likely due to MCFA recycling from TAG and membrane lipids followed by degradation via β-oxidation.	(Knutzon *et al.*, 1999)
Camelina sativa	-	0% MCFA:<br>28-30% seed oil	No MCFA production	(Kim *et al.*, 2015)
	FATB1 from *U. californica*<br><br>FATB1 from *U. californica* and LPAT from *Cocos nucifera*	~19% 12:0;<br>~30% seed oil<br><br>~29% 12:0;<br>~28% seed oil	Target MCFA accumulated but levels are lower than previous engineering in *Brassica*. Species-specific constraints include difference in the pathway fluxes or enhanced 12:0 degradation. Further analysis is required to pinpoint limiting enzymatic or pathway fluxes.	

	FATB1 from *C. viscosissma*	~13% of MCFA (10:0, 12:0, 14:0); ~22% of seed oil	Target MCFAs are accumulated. Co-expression with *CvLPAT2* enhanced incorporation of MCFA into *sn*-2 position of TAG. DGAT1 from *C. pulcherrima* helped incorporate MCFA-CoA into TAG. Nonetheless, accumulation was limited, likely due to difference in DAG pool utilization. Combined expression of MCFA-preferring LPAT and DGAT improved MCFA accumulation to ~30%. This suggests synergistic effects, yet seed oil content is reduced due unknown host metabolic bottlenecks, and/or degradation pathways.	(Iskandarov *et al.*, 2017)
	FATB1 and LPAT2 from *C. viscosissma*	~ 13-14% of MCFA (10:0, 12:0, 14:0); ~19% of seed oil		
	CvFATB1 and DGAT1 from *C. pulcherrima*	~14-18% MCFA (10:0, 12:0, 14:0); ~20% of seed oil		
	FATB1, LPAT2 from *C. viscosissma and* DGAT1 from *C. pulcherrima*	~25-30% MCFA (10:0, 12:0, 14:0); ~22% of seed oil		
Thlaspi arvense	-	0% MCFA; ~30-35% seed oil	No MCFA production	(Esfahanian *et al.*, 2021)
	FATB1 from *U. californica* and LPAT from *Cocos nucifera*	10-14% 12:0; ~26% seed oil	Similar to *Camelina*, MCFA accumulation was achieved, but seed oil was reduced.	
	FATB1 and LPAT2 from *C. viscosissma*	9-11% MCFA (8:0, 10:0, 12:0); ~23% seed oil		
	FATB1, LPAT2 from *C. viscosissma and* DGAT1 from *C. pulcherrima*	~11-12% MCFA (8:0, 10:0, 12:0); ~25% seed oil		
Epoxy fatty acid (Vernolic acid) engineering				
Arabidopsis	Epoxygenase (EPX) from *C. palaestina*	~6.2% of epoxy FA; ND	Vernolic acid produced at low levels due to poor flux into TAG. FAD2 co-expression enhanced vernolic acid synthesis, showing desaturation synergy. Co-expression of epoxy-FA-specific DGATs, especially with VgDGAT2	(Singh *et al.*, 2001)
Arabidopsis fad3/fae1	Epoxygenase (EPX) and FAD2 from *C. palaestina*	~21% of epoxy fatty acids		(Zhou *et al.*, 2006)

Glycine max	-	0% of epoxy FA	improves accumulation. However, efficient flux into TAG, host metabolism may still limits total accumulation.	(Li *et al.*, 2010)
	Epoxygenase (EPX) from *S. laevis*	~8% of epoxy FA; ND		
	SIEPX and DGAT1 from V. galamensis	~15% of epoxy FA; ND		
	SIEPX and DGAT2 from V. galamensis	~26% of epoxy FA; ND		
Conjugated fatty acid (Eleostearic acid) engineering				
Arabidopsis fad3/fae1	FA desaturase/ Conjugase (FADX) from *V. fordii*	~4-10% of Eleostearic acid; ND	Low EA accumulation due to poor flux from membrane lipids to TAG. Co-expression of LPAT2 and DGAT1 increased EA incorporation into TAG, but full redirection from PC remains a challenge.	(Cahoon *et al.*, 2006)
	FADX from M. charantia	13% of Eleostearic acid; ND		
	FADX and LPAT2 from *V. fordii*	~4-5% Eleostearic acid; ND		(Shockey *et al.*, 2019)
	FADX and DGAT2 from *V. fordii*	~5-10% Eleostearic acid; ND		
	FADX, LPAT2 and DGAT2 from *V. fordii*	~5-14% Eleostearic acid; ND		
Cyclopropane fatty acids (CPFAs) engineering				
Arabidopsis fad2/fae1	Cyclopropane synthase (CPS) from *E. coli*	~5-9.1% of CPA; ~35% seed oil	Low amounts of target fatty acids are accumulated in EcCPS alone engineered plants. CPFA accumulates in *sn*-1 position of PC but not efficiently transferred to TAG, likely due to limited conversion of CPFA-PC to DAG and poor removal of CPFA from sn-1 PC, as acyl editing predominantly occurs at the sn-2 position. Co-expression of LPAT or DGATs from different species slightly enhanced the levels of CPFA, but the levels are very low. Suggests LPAT sn-2 and DGATs CPFA-CoA specificity is beneficial but insufficient alone without improving DAG-PC interconversion. PDCT improved CPFA transfer	(Yu *et al.*, 2014)
	EcCPS and LPAT from *S. foetida*	~35% of CPFA; ~30% seed oil		
	EcCPS and DGAT1 from *Gossypium hirsutum* or *L. chinensis*	~10-12% CPFA; ND<br>~10-15% CPFA; ND		(Shockey *et al.*, 2023)
	EcCPS and DGAT2 from *Gossypium*	~8-12% CPFA; ND		

	hirsutum or *L. chinensis*	~12-17% CPFA; ND	from PC to DAG, but high CPFA retention in PC indicates persistent bottlenecks. CPFA specific acyl editing and tuning DAG-PC cycle is needed.	
Camelina sativa	Cyclopropane synthase (CPS) from *E. coli*	~10% of CPFA; ~30% seed oil		(Yu *et al.*, 2019)
	EcCPS and PDCT from *L. chinensis*	~9% CPFA; ~30% seed oil		

References

Bates PD, Browse J. 2011. The pathway of triacylglycerol synthesis through phosphatidylcholine in Arabidopsis produces a bottleneck for the accumulation of unusual fatty acids in transgenic seeds. *The Plant Journal* **68**: 387–399.

Bates PD, Johnson SR, Cao X, Li J, Nam J-W, Jaworski JG, Ohlrogge JB, Browse J. 2014. Fatty acid synthesis is inhibited by inefficient utilization of unusual fatty acids for glycerolipid assembly. *Proceedings of the National Academy of Sciences* **111**: 1204–1209.

Broun P, Somerville C. 1997. Accumulation of Ricinoleic, Lesquerolic, and Densipolic Acids in Seeds of Transgenic Arabidopsis Plants That Express a Fatty Acyl Hydroxylase cDNA from Castor Bean. *Plant Physiology* **113**: 933–942.

Burgal J, Shockey J, Lu C, Dyer J, Larson T, Graham I, Browse J. 2008. Metabolic engineering of hydroxy fatty acid production in plants: RcDGAT2 drives dramatic increases in ricinoleate levels in seed oil. *Plant Biotechnology Journal* **6**: 819–831.

Cahoon EB, Dietrich CR, Meyer K, Damude HG, Dyer JM, Kinney AJ. 2006. Conjugated fatty acids accumulate to high levels in phospholipids of metabolically engineered soybean and Arabidopsis seeds. *Phytochemistry* **67**: 1166–1176.

Dehesh K, Jones A, Knutzon DS, Voelker TA. 1996. Production of high levels of 8:0 and 10:0 fatty acids in transgenic canola by overexpression of *Ch FatB2* , a thioesterase cDNA from *Cuphea hookeriana*. *The Plant Journal* **9**: 167–172.

Demski K, Jeppson S, Stymne S, Lager I. 2022. Phosphatidylcholine:diacylglycerol cholinephosphotransferase's unique regulation of castor bean oil quality. *Plant Physiology* **189**: 2001–2014.

van Erp H, Bates PD, Burgal J, Shockey J, Browse J. **2011**. Castor phospholipid:diacylglycerol acyltransferase facilitates efficient metabolism of hydroxy fatty acids in transgenic Arabidopsis. *Plant Physiology* **155**: 683–693.

Esfahanian M, Nazarenus TJ, Freund MM, McIntosh G, Phippen WB, Phippen ME, Durrett TP, Cahoon EB, Sedbrook JC. **2021**. Generating Pennycress (Thlaspi arvense) Seed Triacylglycerols and Acetyl-Triacylglycerols Containing Medium-Chain Fatty Acids. *Frontiers in Energy Research* **9**: 620118.

Hu Z, Ren Z, Lu C. **2012**. The phosphatidylcholine diacylglycerol cholinephosphotransferase is required for efficient hydroxy fatty acid accumulation in transgenic Arabidopsis. *Plant Physiology* **158**: 1944–1954.

Iskandarov U, Silva JE, Kim HJ, Andersson M, Cahoon RE, Mockaitis K, Cahoon EB. **2017**. A Specialized Diacylglycerol Acyltransferase Contributes to the Extreme Medium-Chain Fatty Acid Content of *Cuphea* Seed Oil. *Plant Physiology* **174**: 97–109.

Kim HJ, Silva JE, Vu HS, Mockaitis K, Nam J-W, Cahoon EB. **2015**. Toward production of jet fuel functionality in oilseeds: identification of FatB acyl-acyl carrier protein thioesterases and evaluation of combinatorial expression strategies in *Camelina* seeds. *Journal of Experimental Botany* **66**: 4251–4265.

Knutzon DS, Hayes TR, Wyrick A, Xiong H, Maelor Davies H, Voelker TA. **1999**. Lysophosphatidic Acid Acyltransferase from Coconut Endosperm Mediates the Insertion of Laurate at the *sn-2* Position of Triacylglycerols in Lauric Rapeseed Oil and Can Increase Total Laurate Levels. *Plant Physiology* **120**: 739–746.

Larson TR, Edgell T, Byrne J, Dehesh K, Graham IA. **2002**. Acyl CoA profiles of transgenic plants that accumulate medium-chain fatty acids indicate inefficient storage lipid synthesis in developing oilseeds. *The Plant Journal* **32**: 519–527.

Li R, Yu K, Hatanaka T, Hildebrand DF. **2010**. *Vernonia* DGATs increase accumulation of epoxy fatty acids in oil. *Plant Biotechnology Journal* **8**: 184–195.

Li-Beisson Y, Shorrosh B, Beisson F, Andersson MX, Arondel V, Bates PD, Baud S, Bird D, Debono A, Durrett TP, *et al.* **2013**. Acyl-lipid metabolism. *Arabidopsis Book* **11**: e0161.

Lu C, Fulda M, Wallis JG, Browse J. **2006**. A high-throughput screen for genes from castor that boost hydroxy fatty acid accumulation in seed oils of transgenic Arabidopsis. *The Plant Journal* **45**: 847–856.

Lunn D, Wallis JG, Browse J. 2019. Tri-Hydroxy-Triacylglycerol Is Efficiently Produced by Position-Specific Castor Acyltransferases. *Plant Physiology* **179**: 1050–1063.

Lunn D, Wallis JG, Browse J. 2022. A multigene approach secures hydroxy fatty acid production in Arabidopsis (MC Suh, Ed.). *Journal of Experimental Botany* **73**: 2875–2888.

Poirier Y, Ventre G, Caldelari D. 1999. Increased Flow of Fatty Acids toward β-Oxidation in Developing Seeds of Arabidopsis Deficient in Diacylglycerol Acyltransferase Activity or Synthesizing Medium-Chain-Length Fatty Acids. *Plant Physiology* **121**: 1359–1366.

Regmi A, Shockey J, Kotapati HK, Bates PD. 2020. Oil-Producing Metabolons Containing DGAT1 Use Separate Substrate Pools from those Containing DGAT2 or PDAT. *Plant Physiology* **184**: 720–737.

Shockey J, Lager I, Stymne S, Kotapati HK, Sheffield J, Mason C, Bates PD. 2019. Specialized lysophosphatidic acid acyltransferases contribute to unusual fatty acid accumulation in exotic Euphorbiaceae seed oils. *Planta* **249**: 1285–1299.

Shockey J, Parchuri P, Thyssen GN, Bates PD. 2023. Assessing the biotechnological potential of cotton type-1 and type-2 diacylglycerol acyltransferases in transgenic systems. *Plant Physiology and Biochemistry* **196**: 940–951.

Singh S, Thomaeus S, Lee M, Stymne S, Green A. 2001. Transgenic expression of a Δ12-epoxygenase gene in Arabidopsis seeds inhibits accumulation of linoleic acid. *Planta* **212**: 872–879.

Smith MA, Moon H, Chowrira G, Kunst L. 2003. Heterologous expression of a fatty acid hydroxylase gene in developing seeds of Arabidopsis thaliana. *Planta* **217**: 507–516.

Snapp AR, Kang J, Qi X, Lu C. 2014. A fatty acid condensing enzyme from Physaria fendleri increases hydroxy fatty acid accumulation in transgenic oilseeds of Camelina sativa. *Planta* **240**: 599–610.

Voelker TA, Hayes TR, Cranmer AM, Turner JC, Davies HM. 1996. Genetic engineering of a quantitative trait: metabolic and genetic parameters influencing the accumulation of laurate in rapeseed. *The Plant Journal* **9**: 229–241.

Yu X-H, Cai Y, Chai J, Schwender J, Shanklin J. **2019**. Expression of a Lychee *PHOSPHATIDYLCHOLINE:DIACYLGLYCEROL CHOLINEPHOSPHOTRANSFERASE* with an *Escherichia coli CYCLOPROPANE SYNTHASE* Enhances Cyclopropane Fatty Acid Accumulation in Camelina Seeds. *Plant Physiology* **180**: 1351–1361.

Yu X-H, Prakash RR, Sweet M, Shanklin J. **2014**. Coexpressing *Escherichia coli* Cyclopropane Synthase with *Sterculia foetida* Lysophosphatidic Acid Acyltransferase Enhances Cyclopropane Fatty Acid Accumulation. *Plant Physiology* **164**: 455–465.

Zhou X-R, Singh S, Liu Q, Green A. **2006**. Combined transgenic expression of Δ12-desaturase and Δ12-epoxygenase in high linoleic acid seeds leads to increased accumulation of vernolic acid. *Functional Plant Biology* **33**: 585.
